# Functional changes of the liver in the absence of growth hormone (GH) action – Proteomic and metabolomic insights from a GH receptor deficient pig model

**DOI:** 10.1016/j.molmet.2020.100978

**Published:** 2020-03-18

**Authors:** Evamaria O. Riedel, Arne Hinrichs, Elisabeth Kemter, Maik Dahlhoff, Mattias Backman, Birgit Rathkolb, Cornelia Prehn, Jerzy Adamski, Simone Renner, Andreas Blutke, Martin Hrabĕ de Angelis, Martin Bidlingmaier, Jochen Schopohl, Georg J. Arnold, Thomas Fröhlich, Eckhard Wolf

**Affiliations:** 1 Laboratory for Functional Genome Analysis, (LAFUGA), Gene Center, LMU Munich, 81377 Munich, Germany; 2 Medizinische Klinik und Poliklinik IV, Klinikum der LMU München, 80336 Munich, Germany; 3 Institute of Molecular Animal Breeding and Biotechnology, Gene Center and Department of Veterinary Sciences, LMU Munich, 81377 Munich, Germany; 4 German Center for Diabetes Research (DZD), 85764 Neuherberg, Germany; 5 German Mouse Clinic (GMC), Institute of Experimental Genetics, Helmholtz Zentrum München, 85764 Neuherberg, Germany; 6 Research Unit Molecular Endocrinology and Metabolism (MEM), Helmholtz Zentrum München, 85764 Neuherberg, Germany; 7 Chair of Experimental Genetics, School of Life Science Weihenstephan, Technische Universität München, 85354 Freising, Germany; 8 Department of Biochemistry, Yong Loo Lin School of Medicine, National University of Singapore, Singapore; 9 Research Unit Analytical Pathology, Helmholtz Zentrum München, 85764 Neuherberg, Germany

**Keywords:** Liver, Growth hormone, Laron syndrome, Pig model, Proteomics, Metabolomics

## Abstract

**Objective:**

The liver is a central target organ of growth hormone (GH), which stimulates the synthesis of insulin-like growth factor 1 (IGF1) and affects multiple biochemical pathways. A systematic multi-omics analysis of GH effects in the liver has not been performed. GH receptor (GHR) deficiency is a unique model for studying the consequences of lacking GH action. In this study, we used molecular profiling techniques to capture a broad spectrum of these effects in the liver of a clinically relevant large animal model for Laron syndrome.

**Methods:**

We performed holistic proteome and targeted metabolome analyses of liver samples from 6-month-old GHR-deficient (*GHR*-KO) pigs and GHR-expressing controls (four males, four females per group).

**Results:**

GHR deficiency resulted in an increased abundance of enzymes involved in amino acid degradation, in the urea cycle, and in the tricarboxylic acid cycle. A decreased ratio of long-chain acylcarnitines to free carnitine suggested reduced activity of carnitine palmitoyltransferase 1A and thus reduced mitochondrial import of fatty acids for beta-oxidation. Increased levels of short-chain acylcarnitines in the liver and in the circulation of *GHR*-KO pigs may result from impaired beta-oxidation of short-chain fatty acids or from increased degradation of specific amino acids. The concentration of mono-unsaturated glycerophosphocholines was significantly increased in the liver of *GHR*-KO pigs without morphological signs of steatosis, although the abundances of several proteins functionally linked to non-alcoholic fatty liver disease (fetuin B, retinol binding protein 4, several mitochondrial proteins) were increased. Moreover, GHR-deficient liver samples revealed distinct changes in the methionine and glutathione metabolic pathways, in particular, a significantly increased level of glycine N-methyltransferase and increased levels of total and free glutathione. Several proteins revealed a sex-related abundance difference in the control group but not in the *GHR*-KO group.

**Conclusions:**

Our integrated proteomics/targeted metabolomics study of GHR-deficient and control liver samples from a clinically relevant large animal model identified a spectrum of biological pathways that are significantly altered in the absence of GH action. Moreover, new insights into the role of GH in the sex-related specification of liver functions were provided.

## Highlights

• A GHR-deficient pig model was used to study consequences of missing GH actions in the liver.
• Proteomics and metabolomics revealed alterations in multiple biochemical pathways.
• Enzymes and metabolites involved in amino acid degradation, urea cycle, and TCA cycle were increased in abundance.
• Alterations in fatty acid beta-oxidation and in the methionine and glutathione metabolic pathways were revealed.
• New insights into the role of GH in sex-related differentiation of liver functions were provided.

## 1 Introduction

Growth hormone (GH) is an important regulator of postnatal growth and has profound effects on metabolism and energy homeostasis (reviewed in [1,2]). In the liver, GH stimulates the synthesis and secretion of insulin-like growth factor 1 (IGF1), accounting for the majority of IGF1 in the circulation [3]. In addition, GH stimulates hepatic glucose production, but the relative contributions of gluconeogenesis and glycogenolysis are still a matter of discussion (reviewed in [1]). GH also plays a major role in hepatic lipid metabolism, including stimulation of uptake and storage, but also secretion of triglycerides (reviewed in [4]). Patients with adult GH deficiency show an increased prevalence of non-alcoholic fatty liver disease (NAFLD), which improves upon GH treatment (reviewed in [5]). Steatosis was also observed in a proportion of patients with growth hormone receptor (GHR) deficiency (Laron syndrome; OMIM reference 262500) [6] and in mice with a liver-specific deletion of the GHR [7]. To clarify whether this was due to deficient GH action or low IGF1 levels, Liu and coworkers [8] restored IGF1 in liver-specific GHR-deficient mice by a liver-specific *Igf1* transgene. The increase in IGF1 did not prevent steatosis, suggesting that this was a direct consequence of the lack of GH action. Interestingly, global GHR deficiency in mice was not associated with steatosis [9].

In spite of numerous studies addressing GH effects on specific metabolic pathways in the liver, a holistic multi-omics analysis of hepatic GH actions has not been performed. We therefore analyzed liver samples of 6-month-old GHR-deficient (*GHR*-KO) pigs [10] and of littermate controls with intact *GHR* alleles (*GHR*^+/-^ or *GHR*^+/+^) by shotgun proteomics and targeted metabolomics. Samples from male and female pigs were analyzed to take sex-specific differences in metabolism and energy homeostasis (reviewed in [11]) into account.

## 2 Materials and methods

### 2.1 Animal model, collection of blood and liver samples

**Figure 1:**
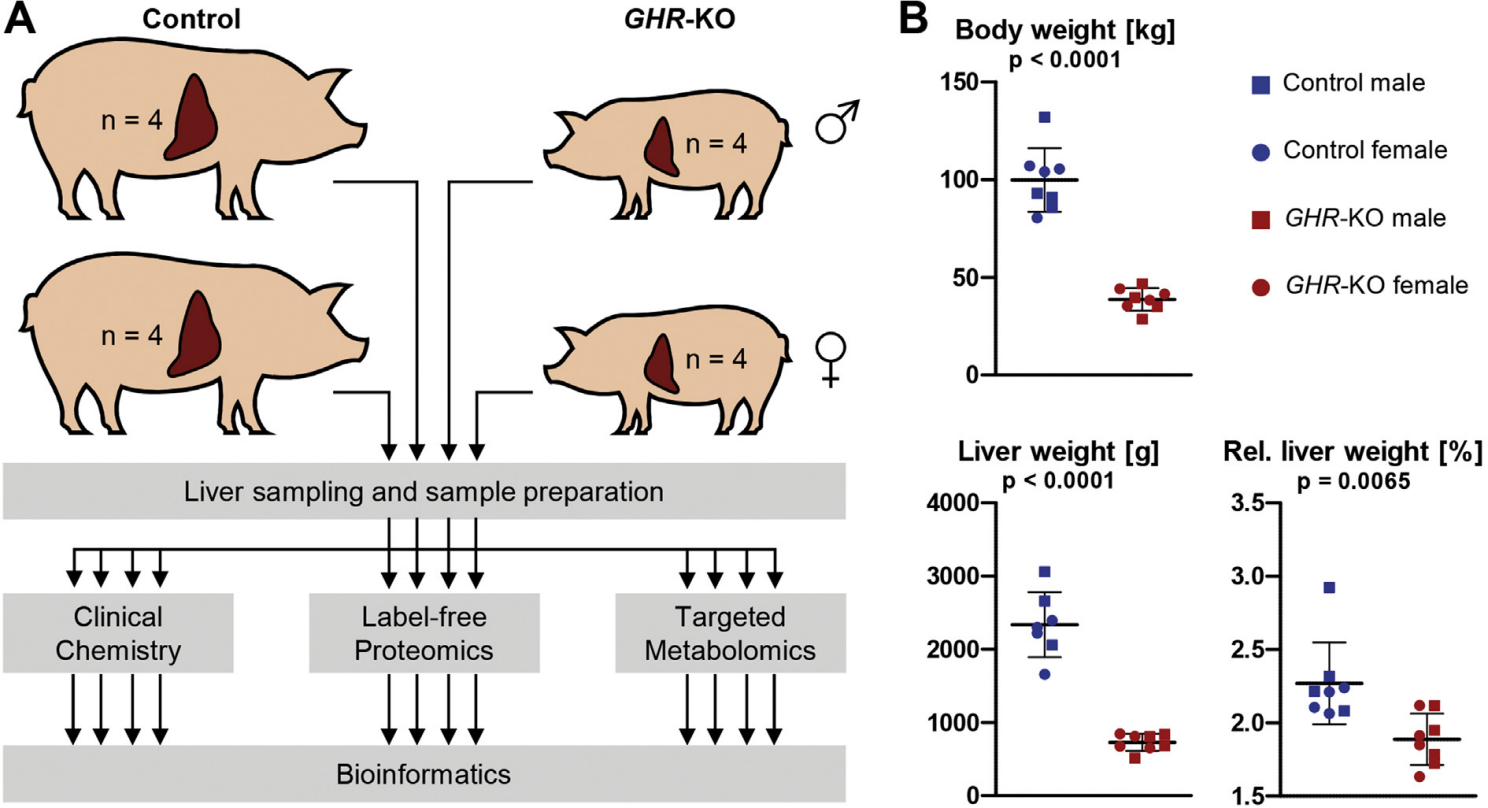
Study outline, body and liver weights. *A:* Serum and liver samples from male and female six-month-old *GHR*-KO and control pigs were analyzed for clinical-chemical parameters, proteins and metabolites affected by group, sex and the interaction group × sex. *B:* Body and liver weights as well as relative liver weights of *GHR*-KO and control pigs.

*GHR*-KO pigs were generated and maintained as described previously [10]. *GHR*-KO pigs (*GHR*^−/-^; four males, four females) and control animals (*GHR*^+/-^, *GHR*^+/+^; two males, two females of each genotype) were euthanized at an age of 6 months after overnight fasting. Since several studies demonstrated circadian oscillations of the liver proteome profile [[12], [13], [14]], necropsies and tissue sampling were always performed at the same time of the day (between 9.00 and 11.00 a.m.). Blood was collected from the jugular vein. After clotting for 30 min at room temperature, serum was separated by centrifugation (1,200 ×g) for 20 min at 6 °C and stored at −80 °C until analysis. Liver samples (100 mg) were taken according to a standardized protocol [15,16], shock frozen on dry ice, and stored at −80 °C until further analysis. The experimental design is summarized in Figure 1A.

### 2.2 Determination of body weight, liver weight and relative body weight

Body and liver weights were determined the same day when pigs were euthanized (at the age of 6 months). Relative liver weight was calculated by dividing liver weight by body weight.

### 2.3 Clinical chemistry

Clinical-chemical parameters in serum were determined using an AU480 autoanalyzer (Beckman–Coulter). For determination of non-esterified fatty acids (NEFA), the NEFA HR kit (Wako Chemicals) was used, and glycerol levels were measured using the test kit GY105 (Randox). Βeta hydroxybutyrate was measured by Alomed (Radolfzell-Böhringen, Germany) using a validated and accredited photometric method. For determination of all other parameters, we used adapted reagents from Beckman–Coulter. Kits and methods have been described in detail by Rathkolb et al. [17].

### 2.4 Proteomics

*Sample preparation:* For sample processing, 15 μl of 8 M urea/0.4 M NH_4_HCO_3_ per mg frozen tissue was added. Tissues were lysed using a homogenizer (ART-MicraD8, ART Prozess- & Labortechnik) twice at a speed of 23,500 rpm for 30 s and centrifuged through QIA-Shredder devices (Qiagen). Protein concentrations were determined by a 660 nm protein assay (Thermo Scientific Pierce) [18] and adjusted with 8 M urea/0.4 M NH_4_HCO_3_ to a concentration of 2 mg/ml. One hundred μg protein was reduced with DTE/TCEP at a concentration of 4.5 mM DTE/2 mM TCEP for 30 min. Cysteine residues were blocked with iodoacetamide (final concentration 8.3 mM) for 30 min in the dark. After adding DTT stock solution to a final concentration of 10 mM, 1:50 (enzyme:substrate) lysyl endopeptidase (Fujifilm Wako) was added to the sample and incubated for 4 h at 37 °C. After dilution with water to a concentration of 1 M urea, 1:50 (enzyme:substrate) porcine trypsin (Promega) was added to the sample and incubated for 18 h at 37 °C.

*Mass spectrometry:* LC-MS/MS was performed on an UltiMate3000 RSLCnano chromatography system (Thermo Scientific) coupled to a Q Exactive HF-X mass spectrometer (Thermo Scientific). One μg of peptides diluted in 0.1% formic acid (FA) was transferred to a trap column (2 cm; Acclaim® PepMap 100, 75 μm × 2 cm, nanoViper C18, 3 μm, 100 Å, Thermo Scientific) and separated at a flow rate of 250 nL/min (Column: 50 cm; PepMap® RSLC, 75 μm × 50 cm, nanoViper C18, 2 μm, 100 Å, Thermo Scientific). LC gradients were consecutive linear gradients from 3% to 25% solvent B (0.1% formic acid, 100% ACN) in 160 min, from 25% to 40% solvent B in 10 min, and from 40% to 85% solvent B in 10 min. For data acquisition, cycles consisted of one full MS scan at a resolution of 60,000 and 15 data-dependent HCD (higher collision dissociation) MS/MS scans at a resolution of 15,000 and a collision energy of 28. Resulting data were evaluated using Thermo Xcalibur Qual Browser. The mass spectrometry proteomics data have been deposited to the ProteomeXchange Consortium via the PRIDE [19] partner repository with the dataset identifier PXD017671.

*Bioinformatics:* For label-free quantification (LFQ) [20], MS data were processed using MaxQuant V1.6.2.10 and the Sus scrofa subset of the NCBI database (NCBI RefSeq Sus scrofa Annotation Release 106 Scrofa 11.1, available at: www.ncbi.nlm.nih.gov/assembly/GCF_000003025.6/). For identification, the following parameters were used: i) Enzyme: Trypsin; ii) Mass tolerance precursor: 10 ppm; iii) Mass tolerance MS/MS: 0.02 Da; iv) Fixed modification: Carbamidomethylation of cysteine; v) Variable modifications: oxidized methionine. FDRs at the peptide and protein level were set to 1%. In case proteins were detected in all replicates of one group but in no replicate of the other group, the MaxQuant imputation feature was used to allow statistical evaluation. Hierarchical clustering was performed in R, a principal component analysis was done with with the Perseus module V1.6.2.2 of MaxQuant. For the DAVID analysis as well as for the discussion, we exclusively used proteins with a p-value < 0.05. To facilitate a meta-analysis addressing also less prominent abundance alterations, the quantitative values (LFQ) of all identified proteins are listed in Supplementary Table 1A. For functional annotation clustering, the DAVID online platform [21] as well as the GlueGO and CluePedia plugins in Cytoscape were used. Furthermore, Gene Set Enrichment Analysis (GSEA) [22] was performed.

### 2.5 Metabolomics

The targeted metabolomics approach was performed using liquid chromatography-electrospray ionization-tandem mass spectrometry (LC-ESI-MS/MS) and flow injection analysis-electrospray ionization tandem mass spectrometry (FIA-ESI-MS/MS) using the AbsoluteIDQ™ p180 Kit (Biocrates Life Sciences AG), which allows for the quantification of 188 metabolites out of 10 μL plasma and liver tissue homogenate [23]. For a list of the 188 quantified metabolites see Supplementary Table 2. Liver tissue samples were processed, extracted, and quantified as described previously [24]. Per mg of frozen liver tissue 3 μL of a dry ice cooled mixture of ethanol/phosphate buffer (85/15 v/v) were added. Sample handling was performed by a Hamilton Microlab STAR™ robot (Hamilton Bonaduz AG) and an Ultravap nitrogen evaporator (Porvair Sciences). An API 4000 triple quadrupole system (Sciex Deutschland GmbH) equipped with a 1200 Series HPLC (Agilent Technologies Deutschland GmbH) and an HTC PAL auto sampler (CTC Analytics) controlled by the software Analyst 1.6.2. were used for mass spectrometric analyses. MultiQuant 3.0.1 (Sciex) and the MetIDQ™ software package were used for data evaluation for the quantification of metabolite concentrations and quality assessment. As reference for the calculation of metabolite concentrations, internal standards were used. Metabolite concentrations in tissue homogenate are given in μM. The LOD (limit of detection) was set to three times the values of the zero samples (PBS). Metabolites were log transformed and Pareto scaled to model them for two-way ANOVA.

### 2.6 Determination of liver glutathione

Total liver glutathione (GSH) and oxidized glutathione (GSSG) concentrations were determined using a Glutathione Colorimetric Detection Kit (EIAGSHC, Invitrogen). Twenty mg of frozen liver tissue was homogenized with a rod homogenizer (Polytron® PT 2500 E) in ice-cold 1 x phosphate-buffered saline (PBS) and immediately centrifuged (14,000 rpm, 10 min, 4 °C). After protein quantification using the Coomassie Plus (Bradford) Assay Kit (Thermo Scientific), the supernatant was deproteinized with 5% 5-sulfo-salicylic acid dihydrate solution (SSA). For GSSG determination, free GSH and other thiols in the samples were blocked with 2-vinylpyridine (2VP). After adding colorimetric detection reagent and reaction mixture provided with the kit, colorimetric reaction was detected at a wavelength of 405 nm using a Tecan Infinite M 200 pro plate reader. Free glutathione concentration was calculated by subtracting GSSG from GSH. Glutathione levels were normalized for protein content and expressed as μmol/g protein.

### 2.7 Immunoblot analyses

Protein samples were separated on 5% stacking/12% separation polyacrylamide gels with a Mini-Protean Tetra Cell (BioRad). Separated proteins were transferred onto polyvinylidene fluoride (PVDF) membranes (0.45 μm, IPVH00010, Millipore) for 30 min at 1.0 A/25 V. Equal loading was assessed by Ponceau S staining. Blots were blocked (5% non-fat dry milk in Tris-buffered saline with 0.1% Tween 20) for 1 h and incubated overnight at 4 °C with the primary antibody (PGC-1α antibody ab54481, GAPDH antibody #2118, Cell Signaling). Detection was performed with horseradish peroxidase-conjugated polyclonal goat anti-rabbit antibody (1:2500, no. 7074, Cell Signaling; for 1 h at RT) and SuperSignal™ West Dura chemiluminescence substrate (Thermo Scientific). Fluorescence imaging was performed using Intas ECL Chemostar. Western blot band intensities were quantitatively analyzed by Adobe Photoshop CS6. Normalized signal intensities of *GHR*-KO and control samples were compared using the Mann–Whitney *U*-test.

### 2.8 Immunohistochemistry

Fixation of liver samples with 4% formalin or methacarn, paraffin embedding and sectioning and histological analyses of liver samples were performed as described previously [25]. Immunohistochemistry on formalin-fixed tissue slices after heat-induced antigen retrieval in citrate buffer (pH 6) was performed using the following primary antibodies: rabbit polyclonal antibody against ARG1 (1:1200, no. 16001-1-AP, proteintech) and rabbit polyclonal antibody against ACADL (1:400, no. 17526-1-AP, proteintech). As secondary antibody, a horseradish peroxidase-conjugated polyclonal goat anti-rabbit antibody (1:200, no. P0448, Dako) was used. Immunohistochemistry on methacarn-fixed tissue slices was performed using rabbit polyclonal antibody against villin-1 (1:100, no. 2369, Cell Signaling) and horseradish peroxidase-conjugated polyclonal goat anti-rabbit antibody (SignalStain Boost IHC Detection Reagent, no. 8114, Cell Signaling). Immunoreactivity was visualized using 3,3-diaminobenzidine tetrahydrochloride dihydrate (DAB) (brown color). Nuclear counterstaining was done with hemalum (blue color).

### 2.9 Statistical analysis

Statistical analyses were performed using R [26] with the qvalue package and SAS (SAS Institute) as well as GraphPad Prism (GraphPad Software). Visualizations were performed using R with the ggplot2 [27] and pheatmap packages and GraphPad Prism. Effects of group (*GHR*-KO, control), sex, and the interaction group × sex were evaluated using two-way ANOVA. If individual clinical-chemical and metabolomics parameters were addressed in a hypothesis-driven manner, student *t*-tests were used.

FDR for the proteomics and metabolomics ANOVA was calculated with the Benjamini-Hochberg procedure using the qvalue package. Values were considered significant at FDR <0.05. Additionally, 2D principal component analysis was performed using the algorithms implemented in Perseus [28]. All figures have a consistent color code (red = *GHR*-KO, blue = control). Protein abundance differences are given as log2 fold change (l2fc).

## 3 Results

### 3.1 Body and liver weights

Body and liver weights of *GHR*-KO pigs were significantly (p < 0.0001) lower than those of controls. In addition, the relative liver weight (liver weight/body weight) was significantly (p = 0.0065) lower in *GHR*-KO vs. control pigs. No sex-related differences were observed for any of these parameters (Figure 1B).

### 3.2 Clinical-chemical and metabolomic findings in serum

**Figure 2:**
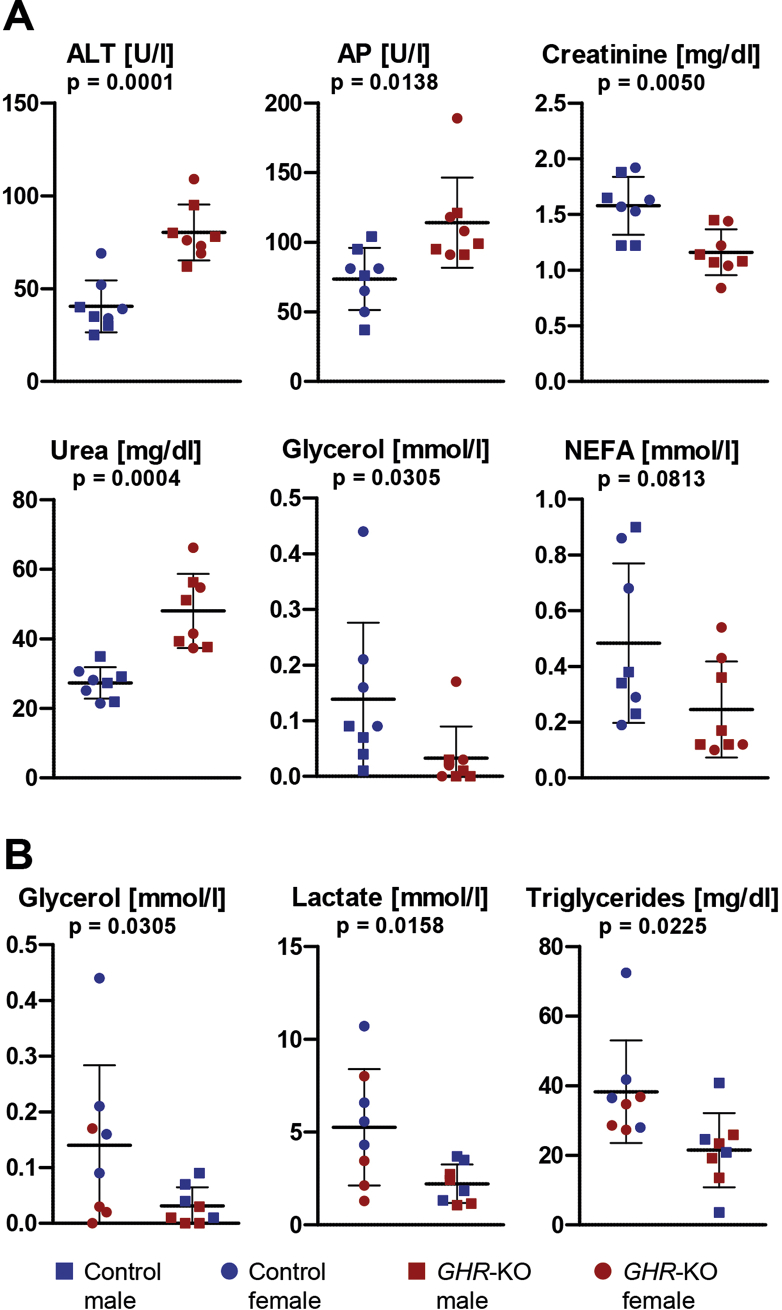
Clinical-chemical parameters in serum. *A:* Parameters affected by group. *B:* Parameters affected by sex. Horizontal lines represent mean and SD. ALT = alanine aminotransaminase, AP = alkaline phosphatase, NEFA = non-esterified fatty acids.

Clinical-chemical analysis of serum samples revealed significantly increased activities of alanine transaminase (ALT; p = 0.0001) and alkaline phosphatase (AP; p = 0.0138) and a higher urea concentration (p = 0.0004) in *GHR*-KO compared to control pigs. The serum levels of creatinine (p = 0.0050) and glycerol (p = 0.0305) were significantly, and those of non-esterified fatty acids (NEFA; p = 0.0813) as a trend lower in *GHR*-KO vs. control samples (Figure 2A). Sex-related differences were observed for the serum concentrations of glycerol (p = 0.0305), triglycerides (p = 0.0225), lactate (p = 0.0158) (Figure 2B) and beta hydroxybutyrate (p = 0.0357), with significantly higher levels in female than in male pigs. The complete set of clinical-chemical data is shown in Supplementary Table 3.

The concentrations of propionylcarnitine, butyrylcarnitine and carnitine were significantly increased in serum samples of *GHR*-KO vs. control pigs. In addition, acetylcarnitine levels were higher (143% of control) in serum samples of *GHR*-KO pigs (Supplementary Figure 1). While not significant by ANOVA, the concentrations of alpha-aminoadipic acid (32% of control), carnosine (74% of control), creatinine (73% of control), spermidine (20% of control), and spermine (11% of control) were reduced in the *GHR*-KO samples (all significant by *t*-test at the p < 0.05 level except for spermidine and spermine due to high variability in the control group).

No sex-related differences were observed for any metabolite concentration in serum, nor was any parameter affected by the interaction group × sex (Supplementary Table 4).

### 3.3 Proteome findings in liver

In total, 32,169 peptides could be identified (PEP-value < 0.05), which could be assigned to 3,231 protein groups at an FDR < 0.01. All 3,231 identified protein groups are – together with a GO analysis via pantherdb.org (Supplementary Figure 2) – listed in Supplementary Table 1A. Only protein groups that were detected in at least five animals of one group (n = 1,913) were selected for further analyses.

*GHR*^+/-^ and *GHR*^+/+^ animals were pooled as a control group, since they were phenotypically not different [10] and also did not show significant differences in the liver proteome profiles (all q-values = 1; Supplementary Table 1B).

**Figure 3:**
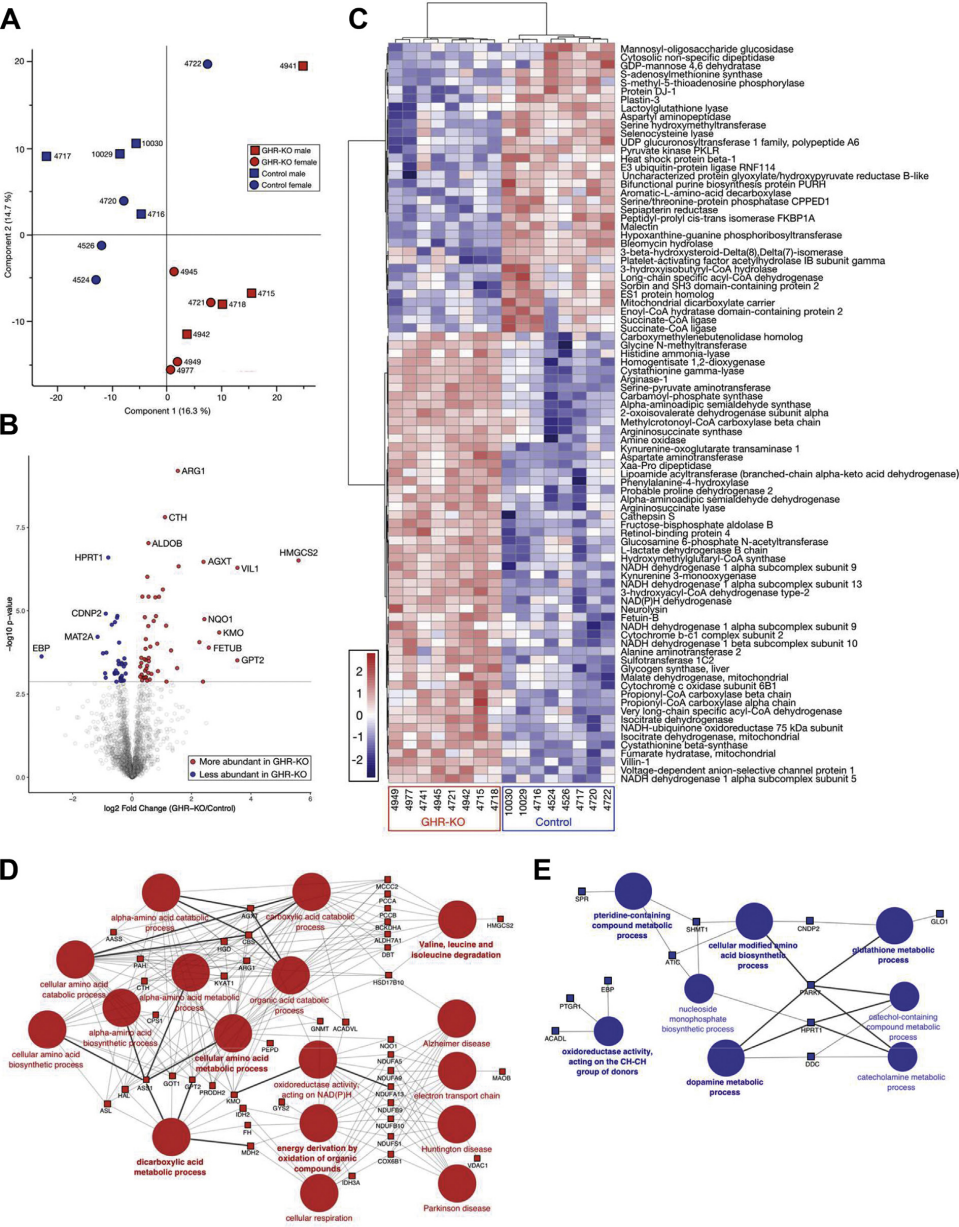
Proteome differences between liver samples from *GHR*-KO and control pigs. *A:* Principal component analysis (PCA) clearly separates proteomes from *GHR*-KO and control samples. *B:* Volcano plot of log2 fold changes (*GHR*-KO/control). Differentially abundant proteins (p < 0.05) are highlighted. *C:* Heat map of the differentially abundant proteins. *D, E:* ClueGO functional enrichment analysis for proteins significantly increased (*D*) or decreased (*E*) in abundance in *GHR*-KO vs. control liver samples. The analysis used the term GO Biological Process and KEGG pathways (min. 8 genes/cluster in *D* and min. 3 genes/cluster in *E*). The size of the circles indicates the significance of enrichment.

A two-dimensional principal component analysis (2D-PCA) clearly separated *GHR*-KO from control pigs (Figure 3A).

Statistical analysis of the proteome data set using a two-way ANOVA (group × sex) identified 87 protein groups that were significantly (p < 0.05) altered in abundance by group, with 53 protein groups (25 with a l2fc > 0.6) being more abundant and 34 protein groups (9 with a l2fc < −0.6) being less abundant in *GHR*-KO vs. control animals (Supplementary Table 5A). A corresponding volcano plot and a heat map are shown in Figure 3B,C.

DAVID analysis of the 53 protein groups with increased abundance in *GHR*-KO pigs revealed three enriched functional clusters, which were related to protein biosynthesis (10 proteins, enrichment score: 6.5), carbon metabolism (especially TCA cycle; 10 proteins, enrichment score: 5.2), and mitochondrial respiration (eight proteins, enrichment score: 4.2).

DAVID analysis of the 34 protein families with decreased abundance in *GHR*-KO pigs showed no enriched functional clusters; most of these proteins are related to metabolism, especially carbohydrate, lipid and amino acid metabolism and to the complement and coagulation cascades. The results of the DAVID analysis are listed in Supplementary Table 5B.

A ClueGO functional enrichment analysis for the significantly more abundant protein groups in the liver of *GHR*-KO vs. control animals in GO Biological Process and KEGG pathways revealed amino acid metabolism, especially amino acid catabolism, dicarboxylic acid metabolism, cellular respiration and electron transport chain as overrepresented (Figure 3D), whereas proteins related to oxidoreductase activity acting on the CH–CH group donors, pteridine-containing compound metabolic process, glutathione metabolic process, dopamine metabolic process, and cellular modified amino acid biosynthetic process were enriched in the set of less abundant proteins (Figure 3E).

In addition, Gene Set Enrichment Analysis (GSEA) [22] was performed using the entire data set of quantified proteins, revealing 206 gene sets significantly enriched in *GHR*-KO pigs and 324 gene sets enriched in control pigs (FDR < 0.25). Gene sets related to mitochondria and the respiratory chain, amino acid metabolism, carbohydrate metabolism, lipid metabolism, oxidoreductase activity, nucleotide/nucleoside metabolism, RNA metabolism, and ribosome were overrepresented in *GHR*-KO pigs, whereas gene sets related to complement and coagulation, immune response and inflammation, protein maturation, modification and localization, regulation of canonical WNT signaling and MAPK pathways, cell structure and regulation of cell death were enriched in control pigs (Supplementary Table 5C).

In addition to genotype-related effects, 16 proteins were significantly altered in abundance by the sex of the animals; 13 proteins were more abundant in male animals, and three proteins were more abundant in female animals. The proteins with the highest sex-related abundance difference were glycine N-phenylacetyltransferase (LOC100517803; 79% homologous to human GLYAT; l2fc 0.85 in males vs. females, p = 0.0033) and dimethylaniline monooxygenase (FMO5; l2fc 1.01 in females vs. males, p = 0.0393). All proteins affected by sex are listed in Supplementary Table 5D.

The abundance of eight proteins was significantly influenced by the interaction group × sex (Supplementary Table 5E). In the control group, the hepatic concentrations of carnosine dipeptidase 2 (CNDP2), proteasome subunit beta 1 (PSMB1), proteasome subunit alpha 4 (PSMA4), aldehyde oxidase 1 (AOX1), flavin containing dimethylaniline monooxygenase 5 (FMO5), and aldehyde dehydrogenase 9 family member A1 (ALDH9A1) were significantly higher in female than in male pigs, whereas the abundance of LOC100517803 (79% homologous to human glycine-N-acyltransferase) was significantly higher in male vs. female pigs. No sex-related difference for the abundance of these proteins was observed in the *GHR*-KO group. Conversely, the level of platelet-activating factor acetylhydrolase 1b catalytic subunit 3 (PAFAH1B3) was significantly higher in female than male *GHR*-KO pigs, whereas no sex-related difference was noted in the control group.

**Figure 4:**
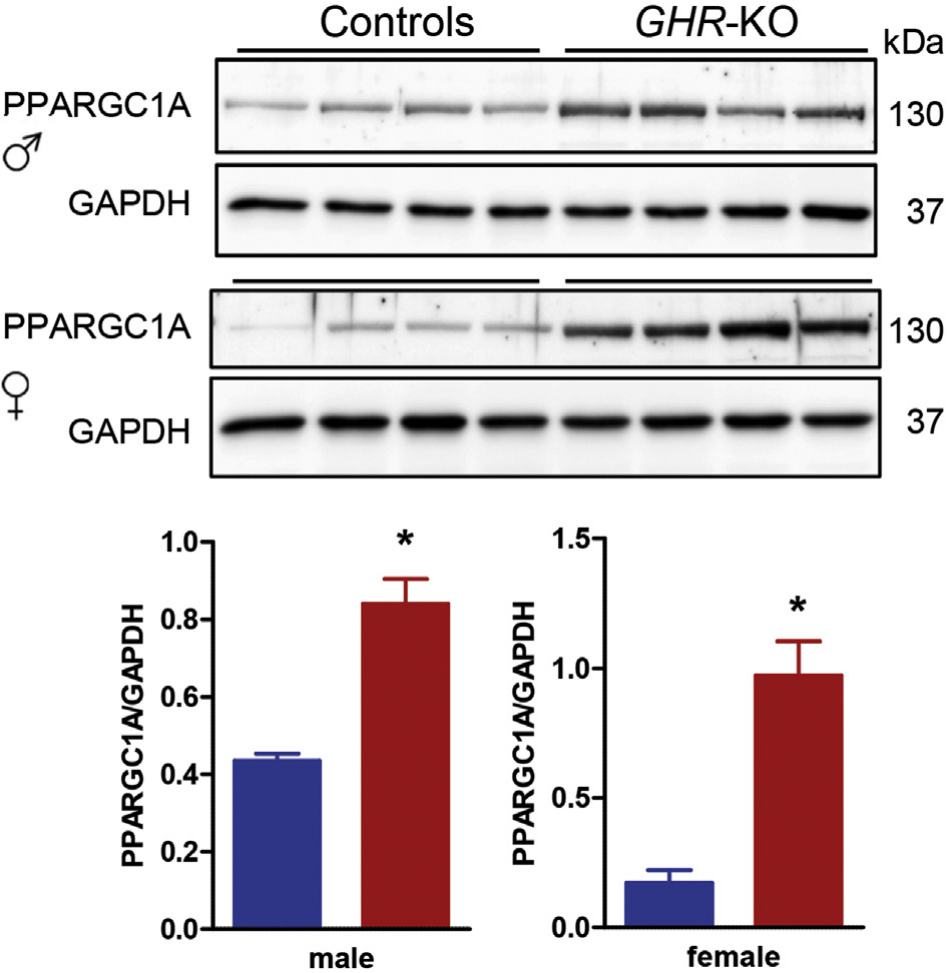
Western blot analysis of PPARGC1A in liver extracts from *GHR*-KO and control pigs. GAPDH was used as reference protein. Corresponding densitometrical analysis illustrating a significantly increased expression of PPARGC1A in liver of *GHR*-KO pigs compared to controls. Data were analyzed by Mann Whitney *U*-test and are presented as means ± SEM (n = 4/group), ∗p < 0.05.

Since the abundance of multiple mitochondrial proteins was increased in liver samples from *GHR*-KO pigs and increased transcript levels of *Ppargc1a* (coding the mitochondrial biogenesis factor PPARG coactivator 1 alpha) were reported in skeletal muscle and kidney tissue from GHR-deficient mice [29], we performed Western blot analyses of PGC-1α (PPARGC1A) in liver extracts from *GHR*-KO and control pigs. Significantly increased levels were detected in both male and female *GHR*-KO pigs compared with sex-matched controls (Figure 4).

**Figure 5:**
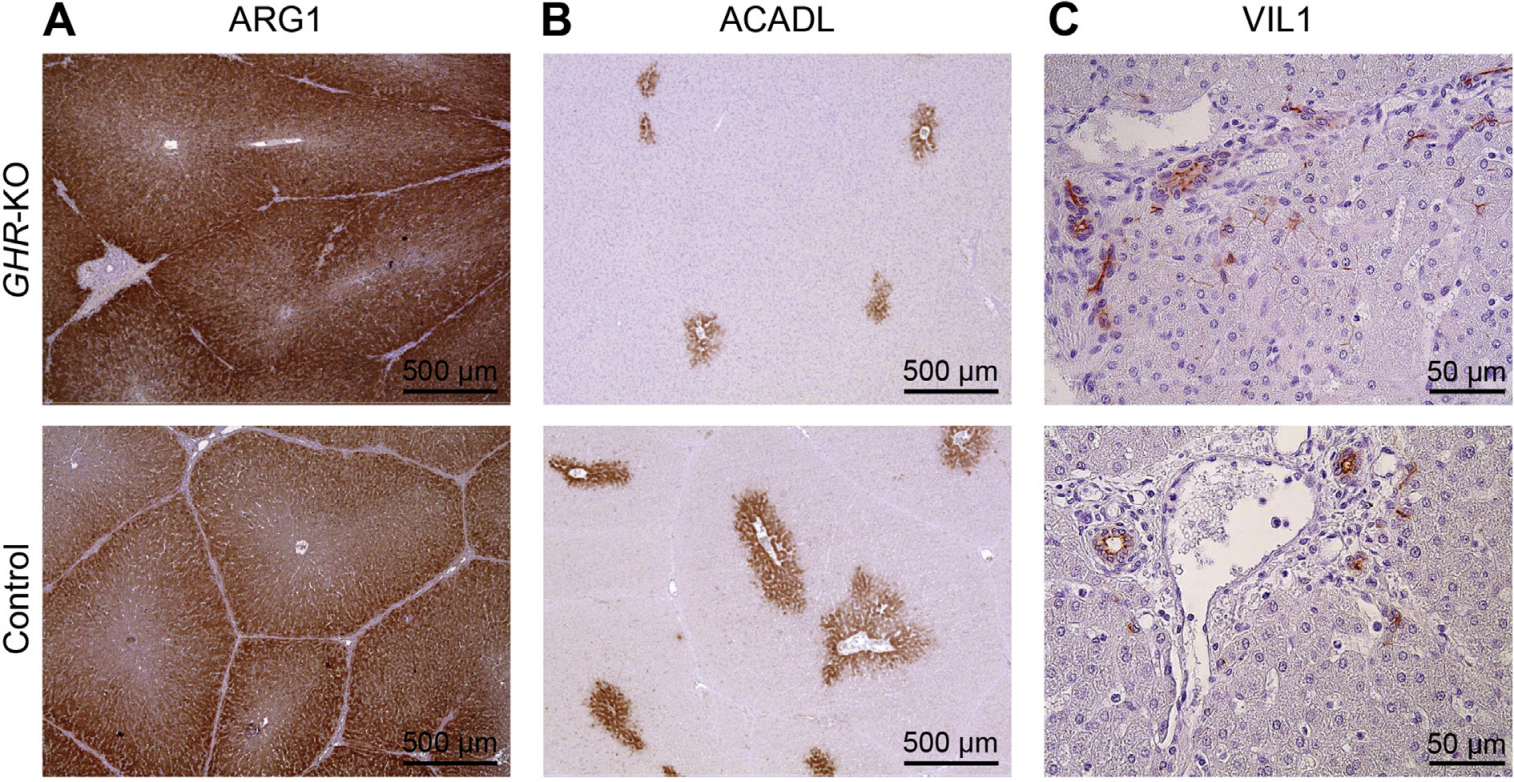
Immunohistochemical localization of arginase 1 (ARG1; *A*), acyl-CoA dehydrogenase long chain (ACADL; *B*), and villin 1 (VIL1; *C*) in liver sections from *GHR*-KO and control pigs.

In addition, we performed localization studies by immunohistochemistry of proteins with major abundance differences between *GHR*-KO and control liver samples and characteristic known expression profiles in different liver zones or cell types. The urea cycle enzyme arginase 1 (ARG1) is predominantly expressed in the periphery of liver lobules [30]. Accordingly, ARG1 staining in control liver samples was stronger in the periphery than in the center of liver lobules. In *GHR*-KO liver samples, which revealed a significantly increased ARG1 concentration (l2fc 1.54; p = 0.26 × 10^−5^), staining was more intensive, but the zonation of the liver lobule was maintained (Figure 5A).

The abundance of acyl-CoA dehydrogenase long chain (ACADL), an enzyme involved in the beta-oxidation of fatty acids, was significantly reduced in *GHR*-KO vs. control liver samples (l2fc −0.91, p = 0.0393). In both groups, centrolobular localization of ACADL was revealed (Figure 5B).

The abundance of villin 1 (VIL1) was markedly increased in the *GHR*-KO liver samples (l2fc 3.55, p = 0.0003). VIL1 staining was revealed at the luminal membrane of bile duct epithelium. In *GHR*-KO samples, some bile canaliculi proximal to the bile ducts were additionally stained (Figure 5C), explaining – at least in part – the higher overall abundance of VIL1 in *GHR*-KO vs. control liver samples.

### 3.4 Liver metabolomics

ANOVA of the liver metabolomics data revealed that the concentrations of five glycerophospholipids (PC aa C32:1, PC aa C32:2, PC aa C34:1, PC ae C34:1, and PC aa C40:4) and of two sphingolipids (SM (OH) C24:1 and SM (OH) C14:1) were significantly higher in *GHR*-KO compared with control liver samples. The same was true for the levels of carnitine (C0), hydroxyvalerylcarnitine (C5–OH/C3-DC-M), and glutarylcarnitine/hydroxyhexanoylcarnitine (C5-DC/C6–OH). While not significant by ANOVA, increased levels of short-chain acylcarnitines, for example, acetylcarnitine (C2; 145% of control), propionylcarnitine (C3; 229% of control), butyrylcarnitine (C4; 176% of control), and valerylcarnitine (C5; 226% of control) were found in the *GHR*-KO liver samples. These differences were all significant (p < 0.05) when testing them individually with student *t*-tests.

**Figure 6:**
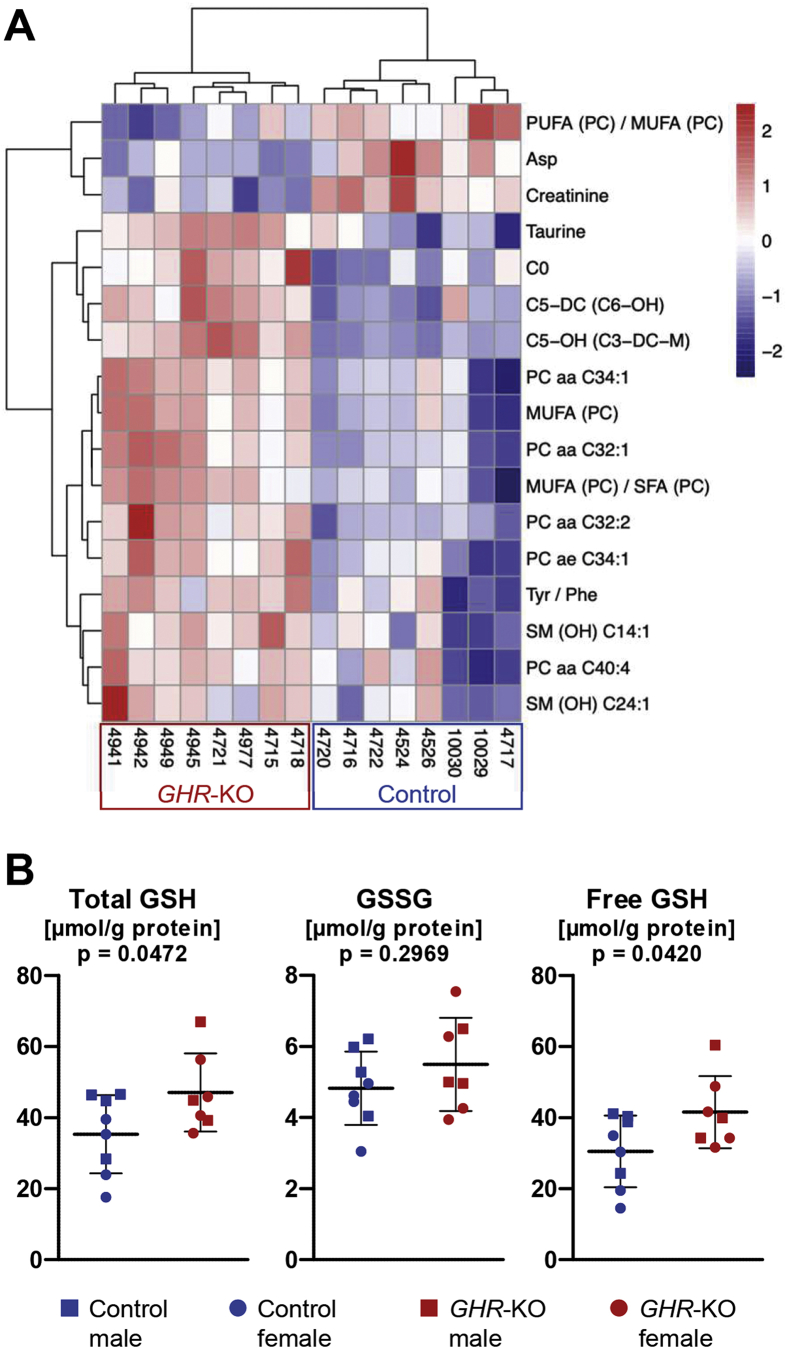
Metabolome differences between liver samples from *GHR*-KO and control pigs. *A:* Heatmap of metabolites significantly affected by group. *B:* Concentrations of total glutathione (GSH), oxidized glutathione (GSSG) and free GSH.

The concentration of mono-unsaturated glycerophosphocholines (MUFA (PC)) and the ratio of mono-unsaturated to saturated glycerophosphocholines (MUFA (PC)/SFA (PC)) were significantly higher, while the ratio of poly-unsaturated to mono-unsaturated glycerophosphocholines (PUFA (PC)/MUFA (PC)) was significantly lower in liver samples of *GHR*-KO vs. control pigs. In addition, the concentration of taurine and the ratio of tyrosine to phenylalanine were significantly increased in the *GHR*-KO samples, whereas the concentrations of creatinine and aspartate were significantly decreased. Furthermore, reduced (*t*-test: p < 0.05) concentrations of the biogenic amines, carnosine (76% of control) and spermine (65% of control), were observed (Figure 6A, Supplementary Figure 3, Supplementary Table 6A).

While no consistent sex-related difference was observed for any metabolite concentration in liver, phenylethylamine, two glycerophospholipids (PC aa C40:4 and PC ae C40:4) and two sphingolipids (SM (OH) C24:1, SM C24:1) were affected by the interaction group × sex (Supplementary Table 6B). In the control group, values for these metabolites were higher in females than in males. In *GHR*-KO pigs, there were no sex-related differences except for phenylethylamine, with higher levels in males than in females.

The complete targeted metabolomics data for liver tissue are shown in Supplementary Table 6C.

In addition, we determined the concentrations of glutathione (GSH) in liver samples from *GHR*-KO and control pigs. The levels of total and free GSH were significantly increased in the *GHR*-KO samples, while the levels of oxidized glutathione (GSSG) were not different between the two groups (Figure 6B, Supplementary Table 6D).

## 4 Discussion

### 4.1 General aspects

Proteome and metabolome profiling of liver tissue from a clinically relevant GHR-deficient large animal model [10] and appropriate controls holds the potential to gain systematic insights into molecular consequences of missing GH action in this central hub of metabolic homeostasis. *GHR*-KO pigs resemble the clinical hallmarks of human Laron syndrome and can be maintained under standardized conditions, thus minimizing variance induced by confounding factors. Moreover, samples can be taken in sufficient amounts according to standardized operating procedures [15], ensuring a sample quality that would be difficult to achieve with human samples. Our experimental design used male and female pigs to comply with the fact that a large proportion of mammalian traits are influenced by sex [31]. Since heterozygous *GHR* mutant (*GHR*^+/-^) pigs were phenotypically and in the liver proteome not different from wild-type (*GHR*^+/+^) animals [10], these two genotypes were pooled as the control group.

Previous proteome studies of GHR-deficient humans or mouse models investigated serum/plasma or white adipose tissue (mouse only) but not liver tissue. Moreover, these studies relied on 2D gel electrophoresis and identification of differentially abundant protein spots by mass spectrometry and revealed only a small number of differentially abundant proteins (reviewed in [32]).

We chose a label-free quantification (LFQ) LC-MS/MS proteomics approach since metabolic labeling, like stable isotope labeling by/with amino acids in cell culture (SILAC) [20], is not applicable for large animal models like the pig. In order to diminish under-sampling, we used long chromatographic gradients (170 min) combined with long separation columns (50 cm, 2 μm beads) instead of pre-fractionation steps, which bear the risk of preventing reliable LFQ-based protein quantification. Retention time stability was controlled thoroughly between each run to ensure the reproducibility of our label-free quantification experiments.

Proteomics of liver tissue is particularly challenging due to the high abundance of several proteins like, for example, plasma proteins; nonetheless we were able to identify 3,231 protein groups at an FDR <1%. A comparison of individual protein abundances in *GHR*^+/-^ and *GHR*^+/+^ pigs using student *t*-tests revealed no protein groups that differed significantly in abundance (q-value of 1, Supplementary Table 1B), indicating that no significant differences between these genotypes were detectable and justifying that they were pooled as control group.

Proteome data were analyzed by ANOVA, taking the effects of group, sex, and the interaction group × sex into account. Liver proteome profiles were mostly influenced by group. Accordingly, a 2D-PCA clearly separated *GHR*-KO and control samples (Figure 3A).

In order to facilitate a comprehensive physiological interpretation of proteome changes in the liver, clinical-chemical analyses of serum and targeted metabolomics of serum and liver tissue were additionally performed.

### 4.2 Increased degradation of amino acids

DAVID GO analysis revealed an annotation cluster including amino acid metabolism and urea cycle as significantly enriched in the *GHR*-KO samples (Supplementary Table 5B), and the gene set enrichment analysis identified many gene sets related to amino acid metabolism as overrepresented (Supplementary Table 5C).

**Figure 7:**
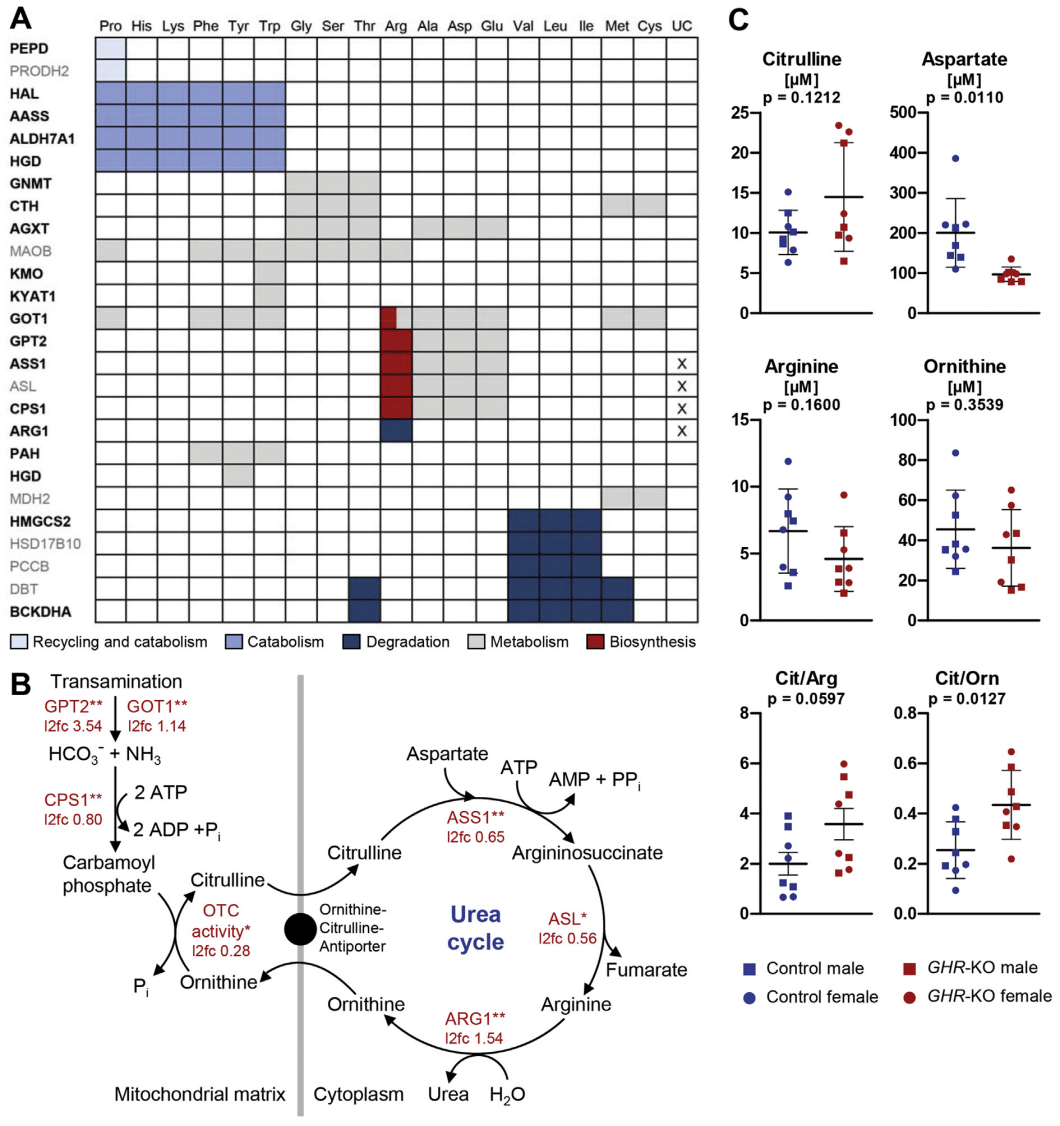
Evidence for increased amino acid degradation in *GHR*-KO vs. control liver samples. *A:* Proteins of amino acid metabolism with significantly (p < 0.05) increased abundance in the *GHR*-KO samples. Black bold font: l2fc > 0.6; grey standard font: l2fc < 0.6. The functions of the enzymes are indicated by color code, X indicates involvement in the urea cycle (UC). *B*: Increased abundance/activity of urea cycle enzymes in *GHR*-KO liver samples. Significant differences are indicated by asterisks: ∗p < 0.05; ∗∗p < 0.01. *C:* Concentrations and ratios of urea cycle substrates. Differences between groups were tested for significance using student *t*-tests. Abbreviations: AASS, alpha-aminoadipic semialdehyde synthase; AGXT, serine-pyruvate aminotransferase; ALDH7A1, alpha-aminoadipic semialdehyde dehydrogenase; ASL, argininosuccinate lyase; ASS1, argininosuccinate synthase 1; ARG1, arginase 1; BCKDHA, 2-oxoisovalerate dehydrogenase subunit alpha; CPS1, carbamoyl-phosphate synthase 1; CTH, cystathionine gamma-lyase; DBT, lipoamide acyltransferase component of branched-chain alpha-keto acid dehydrogenase complex; GNMT, glycine N-methyltransferase; GOT1, aspartate aminotransferase 1; GPT2, alanine aminotransferase 2; HAL, histidine ammonia-lyase; HGD, homogentisate 1,2-dioxygenase; HMGCS2, hydroxymethylglutaryl-CoA synthase; HSD17B10, 3-hydroxyacyl-CoA dehydrogenase type-2; KMO, kynurenine 3-monooxygenase; KYAT1, kynurenine-oxoglutarate transaminase 1; MAOB, amine oxidase; MDH2, malate dehydrogenase; OTC, ornithine carbamoyltransferase; PAH, phenylalanine-4-hydroxylase; PCCB, propionyl-CoA carboxylase beta chain; PEPD, xaa-Pro dipeptidase; PRODH2, probable proline dehydrogenase 2.

The proteome profile of *GHR*-KO liver samples was characterized by significantly increased levels of multiple proteins involved in amino acid metabolism, especially amino acid catabolism. These enzymes and their respective amino acid substrates are illustrated in Figure 7A. In the liver metabolome of *GHR*-KO pigs, the ratio of tyrosine to phenylalanine (Tyr/Phe) was significantly increased (0.78 vs. 0.65 in controls; p = 0.0460), indicating an increased activity of phenylalanine hydroxylase (PAH), the rate-limiting enzyme of the metabolic pathway that degrades excess phenylalanine.

In addition, significantly increased concentrations of glutamic-pyruvic transaminase 2 alias alanine aminotransferase 2 (GPT2; l2fc 3.54, p = 0.0252) and glutamic-oxaloacetic transaminase 1 alias aspartate aminotransferase 1 (GOT1; l2fc 1.14, p = 0.0044) were detected in *GHR*-KO compared to control liver samples. Transamination is an important step in the degradation of amino acids, as it removes their α-amino group and transfers it to α-ketoglutarate. The resulting glutamate undergoes oxidative deamination, releasing ammonia that is detoxified via the urea cycle. The remaining carbon part can be further metabolized in the tricarboxylic acid (TCA) cycle (reviewed in [33]).

All enzymes of the urea cycle, except for ornithine carbamoyltransferase (OTC), were significantly more abundant in *GHR*-KO than in control samples: carbamoyl-phosphate synthase (CPS1; l2fc 0.80, p = 0.0033), argininosuccinate synthase 1 (ASS1; l2fc 0.65, p = 0.0393), argininosuccinate lyase (ASL; l2fc 0.56, p = 0.0232), and arginase 1 (ARG1; l2fc 1.54, p = 0.26 × 10^−5^) (Figure 7B). As an estimate for the activity of these enzymes, we compared the concentrations of citrulline, aspartate, arginine, ornithine, and their ratios (Figure 7C). A significantly reduced concentration of aspartate in *GHR*-KO liver samples may reflect an increased activity of ASS1, using it for the synthesis of argininosuccinate. Another potential explanation is the lower abundance of aspartyl aminopeptidase (DNPEP; l2fc −0.98, p = 0.0180), which releases an N-terminal aspartate or glutamate from peptides, with a preference for aspartate (reviewed in [34]). The trend of lower arginine concentrations in *GHR*-KO samples may be related to reduced synthesis limited by the availability of aspartate or by increased hydrolysis by ARG1. A significantly (p = 0.0127) increased ratio of citrulline to ornithine in the liver metabolome of *GHR*-KO pigs indicates an increased activity of OTC. Significantly increased urea levels in the serum of *GHR*-KO pigs (Figure 2A) underline the increased activity of the urea cycle.

### 4.3 Increased activity of the TCA cycle

**Figure 8:**
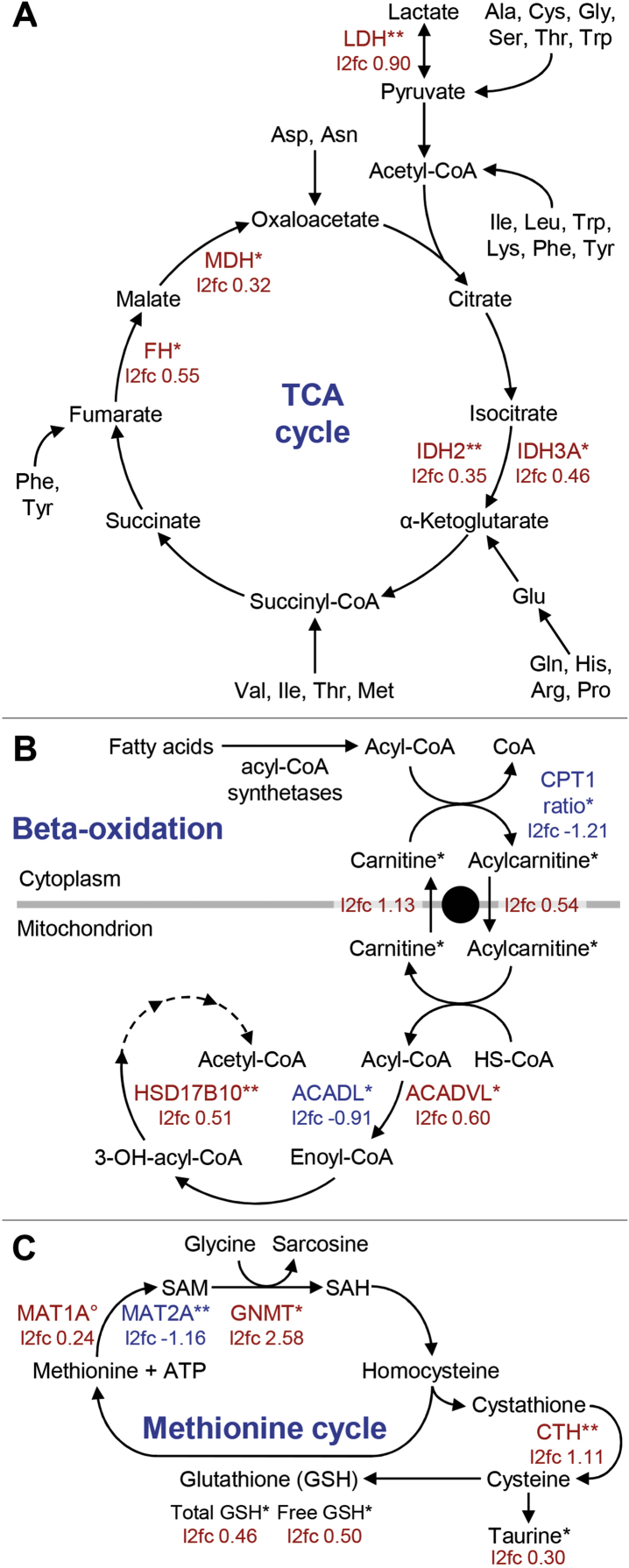
Proteome and metabolome alterations in specific metabolic pathways. Red indicates increased abundance, blue decreased abundance in *GHR*-KO vs. control liver samples. Significant differences are indicated by asterisks: ∗p < 0.05; ∗∗p < 0.01. *A:* Tricarboxylic acid (TCA) cycle. Abbreviations: LDH, lactate dehydrogenase; IDH, isocitrate dehydrogenase; FH, fumarate hydratase; MDH, malate dehydrogenase. *B:* Fatty acid beta-oxidation. Abbreviations: ACAD(V)L, acyl-CoA dehydrogenase (very)long chain; HSD17B10, 3-hydroxyacyl-CoA dehydrogenase type 2; CPT1A, carnitine palmitoyltransferase 1A. CPT1A activity was calculated as ratio of long-chain acylcarnitines to free carnitine. Differences in these parameters were tested for significance using student *t*-tests. *C:* Methionine cycle. Abbreviations: SAM, S-adenosyl-methionine; SAH, S-adenosyl-l-homocysteine; MAT, methionine adenosyltransferases; GNMT, glycine N-methyltransferase; CTH, cystathionine gamma-lyase; ATP, adenosine triphosphate; GSH, glutathione. °p < 0.07.

The carbon part of degraded amino acids is further metabolized, mainly to intermediates of the TCA cycle. The abundance of three TCA cycle enzymes was significantly increased in the *GHR*-KO liver proteome (Figure 8A): isocitrate dehydrogenase (IDH2; l2fc 0.35, p = 0.0012 and IDH3A; l2fc 0.46, p = 0.0446), fumarase (FH; l2fc 0.55, p = 0.0393), and malate dehydrogenase (MDH2; l2fc 0.32, p = 0.046). In addition, a protein cluster related to the TCA cycle and carbon metabolism was identified in the DAVID GO analysis (Supplementary Table 5B), and GSEA revealed 27 proteins related to the KEGG pathway TCA cycle as enriched in the *GHR*-KO samples (Supplementary Table 5C).

Pyruvate or any of the TCA cycle intermediates of glucogenic amino acids can serve as substrates for gluconeogenesis. While GSEA revealed 35 proteins related to the KEGG pathway glycolysis/gluconeogenesis as enriched in the *GHR*-KO samples (Supplementary Table 5C), none of the enzymes involved in gluconeogenesis were significantly altered in abundance in *GHR*-KO vs. control liver samples. However, pyruvate kinase (PKLR, liver and red blood cell isoform), the enzyme catalyzing the last of the three rate-limiting steps of glycolysis, was significantly less abundant in the *GHR*-KO pigs (l2fc −0.89, p = 0.0033). Reduced levels of PKLR may be a mechanism to maintain gluconeogenesis in the absence of GH action by reducing the conversion of phosphoenolpyruvate (PEP) into pyruvate and thus preserving PEP as the basic substrate for gluconeogenesis. A characteristic consequence of GHR deficiency in humans is juvenile hypoglycemia, but glucose levels normalize when the patients become adults [35,36]. Similarly, young (3 months old) *GHR*-KO pigs showed fasting hypoglycemia, whereas normal glucose levels were measured at age 6 months [10]. A common explanation for juvenile hypoglycemia in GHR deficiency is the lack of stimulatory GH effects on hepatic glucose production by glycogenolysis and gluconeogenesis (reviewed in [4]). Different mechanisms, including changes in insulin secretion and insulin sensitivity or increased gluconeogenesis, may account for the normalization of glucose levels during puberty. Future studies determining the rates of gluconeogenesis in young and mature *GHR*-KO pigs along with the levels of gluconeogenic enzymes and potential regulatory proteins in the liver will clarify, if the reduction of PKLR levels in 6-month-old *GHR*-KO pigs indeed has an effect on gluconeogenesis.

The B subunit of lactate dehydrogenase (LDHB) was significantly more abundant in *GHR*-KO vs. control liver tissue (l2fc 0.90, p = 0.0012). LDH, a tetrameric enzyme, catalyzes the interconversion of lactate and pyruvate. Recent studies indicate that lactate can be used as carbon source for the TCA cycle both in tumors and normal tissues (reviewed in [37]). A study of rat liver showed that the LDH isoforms LDHA_4_ (LDH5) and LDHA_3_B (LDH4) are both present in the cytosol and the peroxisomal matrix of hepatocytes, with peroxisomes comprising relatively more LDH-A3B than the cytosol [38]. Intraperoxisomal (and intramitochondrial) LDH may be involved in the re-oxidation of NADH generated by the beta-oxidation pathway (reviewed in [39]).

### 4.4 Marked up-regulation of 3-hydroxy-3-methylglutaryl-CoA synthase 2

The protein with the highest abundance increase in *GHR*-KO vs. control liver tissue was 3-hydroxy-3-methylglutaryl-CoA synthase 2 (HMGCS2; l2fc 5.60, p = 0.0002), the key enzyme of ketogenesis (reviewed in [40]). The expression of HMGCS2 is subject to complex regulation, involving hormones, nutrient-responsive pathways, and posttranslational modifications (reviewed in [[40], [41], [42]]).

For instance, insulin suppresses *HMGCS2* expression via activation of the PI3K-AKT pathway leading to inactivation by phosphorylation and nuclear export of the forkhead box transcription factor FOXA2 (reviewed in [40]). Consequently, high levels of HMGCS2 have been observed in the liver of a genetically engineered pig model for insulin-deficient diabetes mellitus [43]. A direct role of GH in the regulation of HMGCS2 has not, to our knowledge, been described so far. A potential link between deficient GH action and highly upregulated HMGCS2 are increased levels of peroxisome proliferator-activated receptor-gamma (PPARG), which were previously described in the liver of *GHR*-KO pigs [10]. A stimulatory role of PPARG on HMGCS2 expression has been observed, for example, in a mouse model of diabetic cardiomyopathy [44] and in intestinal tumor cell lines and normal intestinal organoids [45]. Increased levels of PPARG in the *GHR*-KO liver samples may also be responsible for the higher abundance of glycogen synthase 2 (GYS2; l2fc 1.16, p = 0.0497), the rate limiting enzyme in the storage of glycogen in liver and adipose tissue [46].

In spite of the marked up-regulation of the key ketogenic enzyme HMGCS2 in *GHR*-KO liver, there was no evidence for stimulated ketogenesis. First, the levels of beta hydroxybutyrate in serum were not different between *GHR*-KO and control pigs. Second, the concentrations of ketogenic amino acids in the circulation and in the liver were not different between *GHR*-KO and control pigs. Third, the serum levels of non-esterified fatty acids, the main substrate of ketogenesis, were, as a trend, reduced in *GHR*-KO pigs, reflecting the missing lipolytic action of GH that also resulted in significantly reduced serum levels of glycerol.

### 4.5 Reduced mitochondrial uptake of fatty acids for beta-oxidation

After cellular uptake of fatty acids via different mechanisms, they are converted to acyl-CoA by acyl-CoA synthetases (reviewed in [47]). Several isoforms of acyl-CoA synthetases were detected in our proteome study, but their abundances were not significantly different between *GHR*-KO and control liver samples. Since the mitochondrial membrane is impermeable for acyl-CoAs, they are converted into acylcarnitines by carnitine palmitoyltransferase 1A (CPT1A). A proxy for the activity of this carnitine shuttle is the ratio of long-chain acylcarnitines (C16 + C18) to free carnitine (C0) (CPT1 ratio). This ratio was lower in *GHR*-KO compared to control animals both in the liver (0.0060 ± 0.0041 vs. 0.0139 ± 0.0071; p = 0.0197) and in the serum (0.0045 ± 0.0025 vs. 0.0079 ± 0.0016; p = 0.0078), indicating a reduced mitochondrial uptake of fatty acids for beta-oxidation. In contrast, the concentration of C0 was significantly higher in the *GHR*-KO than in the control liver samples. In humans, 75% of carnitine is obtained from the diet, whereas the remaining proportion is synthetized from the essential amino acids lysine and methionine in liver, kidney and brain (reviewed in [48]). Since *GHR*-KO and control pigs received the same standardized diet, the higher C0 levels in *GHR*-KO pigs are most likely due to increased endogenous C0 synthesis, probably associated with the generally increased amino acid metabolism (see above).

The abundances of several enzymes involved in fatty acid beta-oxidation were significantly different between *GHR*-KO and control pigs. While acyl-CoA dehydrogenase long chain (ACADL; l2fc −0.91, p = 0.0393) was less abundant in the *GHR*-KO samples, acyl-CoA dehydrogenase very long chain (ACADVL; l2fc 0.60, p = 0.0483) and 3-hydroxyacyl-CoA dehydrogenase type 2 (HSD17B10; l2fc 0.51, p = 0.0004) were more abundant (Figure 8B). Interestingly, increased concentrations of short-chain acylcarnitines were found in liver (C2, C3, C4, C5, C5–OH/C3-DC-M, C5-DC/C6–OH) and serum (C2, C3, C4) of *GHR*-KO pigs. In the context of reduced serum levels of non-esterified fatty acids and reduced mitochondrial up-take of long-chain fatty acids (reduced activity of CPT1A, see above), increased short-chain acylcarnitine levels may result from an impairment of beta-oxidation of short-chain fatty acids. In addition, the degradation of lysine, tryptophan, valine, leucine, and isoleucine can yield short-chain acylcarnitines (C3 and C5 and other carnitines) (reviewed in [49]). We observed an increased amino acid degradation (see above). Increased concentrations of short-chain acylcarnitines were reported to have positive effects in terms of reversing age-dependent deficits in cellular function by improving energy balance (reviewed in [50]).

### 4.6 Increased synthesis of mono-unsaturated fatty acids and other changes in lipid metabolism

In the liver of *GHR*-KO pigs, the concentration of mono-unsaturated glycerophosphocholines (MUFA (PC)) was significantly increased, whereas the concentrations of saturated (SFA (PC)) and poly-unsaturated (PUFA (PC)) glycerophosphocholines were not different from control samples. Consequently, the ratio MUFA (PC)/SFA (PC) was significantly (p = 0.0290) increased, whereas the ratio PUFA (PC)/MUFA (PC) was significantly (p = 0.0353) decreased in the *GHR*-KO samples. MUFA can either be obtained from the diet, or be synthesized by elongase and desaturase enzymes from SFA (reviewed in [51]). Since *GHR*-KO and control pigs received the same standard diet as controls, increased synthesis is the most likely cause of significantly increased MUFA (PC) concentrations in GHR-deficient liver samples. The enzyme catalyzing the critical committed step in the *de novo* synthesis of MUFA is stearoyl-coenzyme A desaturase (SCD). Our holistic proteome analysis did not detect SCD in control liver samples (0/8), but in 3/8 samples from *GHR*-KO pigs. A direct effect of GH on the expression of SCD has been demonstrated in an immunodeficient, liver-damaged mouse model whose liver was almost completely repopulated with human hepatocytes. Since human hepatocytes do not respond to rodent GH, they were in a GH-deficient state. Upon treatment of the mice with human GH, the human *SCD* transcript level was significantly reduced compared to the GH-deficient state [52].

SCD requires NADH, the flavoprotein cytochrome b5 reductase, and the electron acceptor cytochrome b5 as well as molecular oxygen to introduce a single double bond in a spectrum of methylene-interrupted fatty acyl-CoA substrates. NADH is provided by the TCA cycle, which appears to be more active in *GHR*-KO than in control liver (see above). The preferred substrates of SCD are palmitoyl- and stearoyl-CoA, which are then converted into palmitoleoyl- and oleoyl-CoA, respectively. These products are the most abundant MUFA and serve as substrates for the synthesis of phospholipids, triglycerides, cholesteryl esters, and alkyldiacylglycerols (reviewed in [53]). While steatosis has been described in some patients with Laron syndrome [6] and in male mice with liver-specific GHR deficiency [7,54], this was not observed in mice with global GHR deficiency [9] nor in our *GHR*-KO pig model. Nevertheless, the abundances of fetuin B (FETUB; l2fc 2.39, p = 0.0497), a hepatokine increased in patients with liver steatosis [55], of retinol binding protein 4 (RBP4; l2fc 0.73, p = 0.0038) that stimulates hepatic lipogenesis [56], and of several mitochondrial proteins (see below), which DAVID functional annotation clustering links to NAFLD, were significantly increased in the *GHR*-KO liver samples. It is worth noting that pigs have a natural resistance to steatosis, even in morbid obesity [57].

Interestingly, the abundance of solute carrier family 25 member 10 (SLC25A10), a mitochondrial dicarboxylate carrier that plays an important role in supplying malate for citrate transport required for fatty acid synthesis [58], was significantly decreased in the *GHR*-KO liver samples (l2fc −0.49, p = 0.0393). Furthermore, 3-beta-hydroxysteroid-delta(8),delta(7)-isomerase alias EBP cholestenol delta-isomerase (EBP), an endoplasmic reticulum membrane protein that catalyzes the conversion of delta(8)-sterols into delta(7)-sterols and is involved in cholesterol biosynthesis [59], was markedly decreased in abundance in *GHR-*KO vs. control liver tissue (l2fc −3.05, p = 0.0218).

### 4.7 Increased abundance of multiple mitochondrial proteins

Several subunits of the mitochondrial membrane respiratory chain NADH dehydrogenase (Complex I) were more abundant in the liver of *GHR*-KO animals: the core subunit S1 (NDUFS1; l2fc 0.43, p = 0.0033), the alpha subcomplex subunits A5 (NDUFA5; l2fc 0.44, p = 0.0373), A9 (NDUFA9; l2fc 0.40, p = 0.0446) and A13 (NDUFA13; l2fc 0.55, p = 0.0044), and the beta subcomplex subunits B9 (NDUFB9; l2fc 0.42, p = 0.0241) and B10 (NDUFB10; l2fc 0.34, p = 0.0497). In addition, DAVID GO analysis found the cellular component (CC) term mitochondrial respiratory chain complex I as enriched in the *GHR*-KO samples (Supplementary Table 5B), and GSEA analysis revealed several terms related to the mitochondrial respiratory chain as enriched. Furthermore, the abundances of cytochrome c oxidase subunit 6B (COX6B, Complex IV; l2fc = 0.48, p = 0.0340) and of cytochrome b-c1 complex subunit 2 (LOC100524613, 94% homologous to human UQCRC2; l2fc 0.27, p = 0.0309) were significantly increased in the liver of *GHR*-KO pigs.

In addition to proteins of the respiratory chain complexes, many other mitochondrial proteins were more abundant in the *GHR*-KO than in the control liver samples, including HMGCS2, GPT2 and GOT1, isocitrate dehydrogenase (NADP(^+^)) 2, mitochondrial (IDH2) and IDH3A, kynurenine 3-monooxygenase (KMO), aminoadipate-semialdehyde synthase (AASS), ACADVL, proline dehydrogenase 2 (PRODH2), fumarate hydratase (FH), hydroxysteroid 17-beta dehydrogenase 10 (HSD17B10), monoamine oxidase B (MAOB), dihydrolipoamide branched chain transacylase E2 (DBT), neurolysin (NLN), and voltage-dependent anion-selective channel protein 1 (VDAC1).

Gesing et al. [29] investigated transcript levels of genes known to be involved in mitochondrial biogenesis in several tissues of GHR-deficient and control mice. The authors reported increased transcript levels of *Ppargc1a* (coding PPARG coactivator 1 alpha, PPARGC1A) in skeletal muscle, of *Prkaa* (coding AMP-activated protein kinase), *Sirt1* (coding sirtuin 1), *Sirt3*, *Nos3* (coding endothelial nitric oxide synthase) and *Mfn2* (coding mitofusin 2) in heart, and of *Ppargc1a*, *Prkaa*, *Sirt3*, *Nos3* and *Mfn2* in kidney of GHR-deficient mice. In our holistic proteome study of liver samples, only SIRT3 and MFN2 were detected at the protein level; however, the signal intensities were too low to allow a reliable quantification of these proteins. Western blot analysis revealed significantly increased concentrations of PPARGC1A in the *GHR*-KO liver samples (p = 0.0286 for both males and females). Nicotinamide phosphoribosyltransferase (NAMPT), a rate-limiting enzyme in mammalian nicotinamide adenine dinucleotide (NAD)^+^ biosynthesis and regulator of SIRT1 that activates PPARGC1A through deacetylation at specific lysine residues (reviewed in [29]), was also more abundant in *GHR*-KO liver samples, although the difference was not statistically significant (l2fc 0.90, p = 0.2575).

### 4.8 Altered methionine and glutathione metabolism

Proteomics of *GHR*-KO and control liver samples revealed abundance alterations of enzymes of the methionine cycle (Figure 8C), which plays a critical role in the regulation of concentrations of S-adenosyl-methionine (SAM), the major biological methyl group donor. SAM is synthesized from methionine and adenosine triphosphate (ATP) by methionine adenosyltransferases (MAT). The abundance of MAT1A, the major enzyme responsible for SAM synthesis in adult liver [60], was slightly higher in *GHR*-KO vs. control samples, whereas MAT2A that is expressed in non-parenchymal cells of the liver (hepatic stellate and Kupffer cells) and extrahepatic tissues (reviewed in [61]) was less abundant (l2fc −1.16, p = 0.0080). In contrast, the major hepatic SAM utilizing enzyme, glycine N-methyltransferase (GNMT), that converts SAM (along with glycine) to S-adenosyl-l-homocysteine and sarcosine, was significantly more abundant in the *GHR*-KO samples (l2fc 2.58, p = 0.0134). Notably, GH-deficient Ames dwarf mice revealed significantly increased MAT and GNMT levels [62] that were reduced upon GH treatment [63].

Increased GNMT levels in *GHR*-KO liver may decrease the availability of SAM and increase downstream products of the methionine cycle, such as homocysteine, cystathionine, and glutathione (reviewed in [64,65]). Cystathionine gamma-lyase (CTH), which converts cystathionine into cysteine, the amino acid limiting glutathione synthesis, was more abundant in the *GHR*-KO samples (l2fc 1.11, p < 0.0001). Indeed, increased levels of free and total glutathione were revealed in the *GHR*-KO liver samples. Cysteine is also a precursor of taurine, which was more abundant in the *GHR*-KO vs. control liver samples. Lactoylglutathione lyase alias glyoxalase I (GLO1) that catalyzes the conversion of hemimercaptal, formed from methylglyoxal and glutathione, to S-lactoylglutathione was reduced (l2fc −0.28; p = 0.0309).

### 4.9 Additional proteome alterations associated with GHR deficiency

The abundance of villin 1 (VIL1) was markedly increased in the *GHR*-KO liver samples (l2fc 3.55, p = 0.0003). Expression of VIL1 in adult pig liver [66] and in normal human liver [67] was described to be restricted to bile canaliculi. Our immunohistochemical analysis detected VIL1 in the luminal membrane of bile duct epithelium in liver samples of both groups. In *GHR*-KO samples, some bile canaliculi proximal to the bile ducts were additionally stained. The markedly increased level of VIL1 in the *GHR*-KO liver samples suggests that GH directly or indirectly suppresses the expression of VIL1 in the bile canaliculi.

Another protein with increased abundance in *GHR*-KO liver samples was sulfotransferase 1C2 (SULT1C2; l2fc 1.52, p = 0.0333). Sulfotransferase enzymes catalyze the sulfate conjugation of hormones, drugs, and xenobiotic compounds, making them more hydrophilic such that they are readily excreted in the urine or bile (reviewed in [68]). The role of GH in the regulation of SULT1C2 has not been described as of yet.

The abundances of hypoxanthine-guanine phosphoribosyltransferase (HPRT; l2fc −0.80, p = 0.0002) and of malectin (MLEC; l2fc −0.68, p = 0.0043) were significantly reduced in the *GHR*-KO samples. HRPT catalyzes the conversion of hypoxanthine to inosine monophosphate and of guanine to guanosine monophosphate, thus playing a central role in the generation of purine nucleotides through the purine salvage pathway [69]. MLEC is a membrane-anchored ER protein that preferentially binds misfolded proteins and directs them to the ER-associated degradation (ERAD) pathway (reviewed in [70]). Neither HPRT nor MLEC has as yet been shown to be affected by alterations in the GH/IGF1 system.

### 4.10 GHR deficiency partially ameliorates sex-related protein abundance differences in the liver

ANOVA revealed that the abundances of eight proteins were significantly influenced by the interaction group × sex (Supplementary Table 5E), that is, they were influenced by sex in one group but not in the other. Seven proteins showed a sex-related abundance difference in the control group but not in *GHR*-KO pigs. In the latter, only one protein was different in abundance between males and females without a sex-related difference in the control group. These findings point to sex-related GH effects on the abundances of specific proteins, which are eliminated in the absence of the GHR.

A sexual dimorphism in the GH secretion pattern has been described in rodents: in male rats, GH secretion is pulsatile, whereas in female rats it is more continuous (reviewed in [71]). Elimination of GH by hypophysectomy abolished the sex specificity of approximately 90% of 1032 genes that were found to be expressed in a sex-dependent manner in rat liver, demonstrating a dominant role of GH in regulating liver sexual dimorphism [72]. Permanent overexpression of GH in male transgenic mice or continuous infusion of GH by mini-pumps into male mice resulted in a “feminization” of liver functions in terms of reducing the synthesis of major urinary proteins (MUPs), which are normally much more abundant in male than in female mice [73].

Sex-dependent differences in GH secretion have also been described in humans. Adult women have more uniform GH pulses throughout the day, while men have a large nocturnal pulse and relatively low GH output over the rest of the day [74]. A more recent study dissecting factors affecting GH secretion found that women younger than 50 years had a 2-fold higher basal, pulsatile, and total GH secretion compared with men in the same age range, but the sex differences for pulsatile and total GH secretion were no longer significant in subjects older than 50 years [75].

In pigs, a comprehensive characterization of the sex-specific patterns of GH secretion has not, to our knowledge, been conducted. Our observation that significant abundance differences in several proteins in the porcine liver were eliminated by ablation of GHR suggests sex-related differences in GH secretion influencing liver functions also in this species.

## 5 Conclusions

Our integrated proteomics/targeted metabolomics study of GHR-deficient and control liver samples from a clinically relevant large animal model identified a spectrum of biological pathways that are significantly altered in the absence of GH action. In particular, enzymes involved in amino acid degradation, in the urea cycle, and in the tricarboxylic acid cycle were increased in abundance. A decreased ratio of long-chain acylcarnitines to free carnitine suggested reduced mitochondrial import of fatty acids for beta-oxidation. The concentration of mono-unsaturated glycerophosphocholines was significantly increased without morphological signs of steatosis. Distinct abundance changes of enzymes in the methionine and glutathione metabolic pathways were associated with increased levels of total and free glutathione. Moreover, new insights into the role of GH in the specification of sex-related liver functions were provided.

The data set generated in this study is an interesting resource for meta-analyses with human and murine data sets that aim, for example, to investigate why GHR-deficient humans and rodents develop steatosis while pigs don't. Another interesting topic are age-related changes in the function of the GH/GHR system, in particular the question of why GHR deficiency leads to juvenile hypoglycemia that normalizes during puberty. Finally, it will be interesting to see to what extent altered liver functions in GHR deficiency are corrected by IGF1 treatment or somatic *GHR* gene therapy.

## Funding

This study was supported in part by a grant from the German Federal Ministry of Education and Research (BMBF) to the German Center for Diabetes Research (DZD) and – in part – by the Deutsche Forschungsgemeinschaft (TRR127).

## Author contributions

E.O.R., T.F. and E.W. conceived the experiments. E.O.R. and E.W. wrote the manuscript. T.F, G.J.A., M.Bi. and J.S. revised the manuscript. All authors contributed to the manuscript and read and approved the final version.

A.H., M.D., and E.W. developed the animal model, A.H. and A.B. collected the samples, E.O.R. and T.F. conducted and analyzed the proteomics measurements. C.P. and J.A. performed the targeted metabolomics analyses, M.Ba. performed bioinformatics analyses on the metabolomics measurements. E.K. performed immunohistochemical studies, S.R. glutathione measurements, B.R. and M.H.d.A. the clinical-chemical measurements, and M.D. the Western blot analysis.

E.O.R., T.F. and E.W. are the guarantors of this work and, as such, had full access to all the data in the study and take responsibility for the integrity of the data and the accuracy of the data analysis.

## References

[1] Vijayakumar A., Yakar S., Leroith D. (2011). The intricate role of growth hormone in metabolism. Frontiers in Endocrinology (Lausanne).

[2] Ranke M.B., Wit J.M. (2018). Growth hormone - past, present and future. Nature Reviews Endocrinology.

[3] Yakar S., Liu J.L., Stannard B., Butler A., Accili D., Sauer B. (1999). Normal growth and development in the absence of hepatic insulin-like growth factor I. Proceedings of the National Academy of Sciences of the U S A.

[4] Vijayakumar A., Novosyadlyy R., Wu Y., Yakar S., LeRoith D. (2010). Biological effects of growth hormone on carbohydrate and lipid metabolism. Growth Hormone & IGF Research.

[5] Takahashi Y. (2017). The role of growth hormone and insulin-like growth factor-I in the liver. International Journal of Molecular Sciences.

[6] Laron Z., Ginsberg S., Webb M. (2008). Nonalcoholic fatty liver in patients with Laron syndrome and GH gene deletion - preliminary report. Growth Hormone & IGF Research.

[7] Fan Y., Menon R.K., Cohen P., Hwang D., Clemens T., DiGirolamo D.J. (2009). Liver-specific deletion of the growth hormone receptor reveals essential role of growth hormone signaling in hepatic lipid metabolism. Journal of Biological Chemistry.

[8] Liu Z., Cordoba-Chacon J., Kineman R.D., Cronstein B.N., Muzumdar R., Gong Z. (2016). Growth hormone control of hepatic lipid metabolism. Diabetes.

[9] Piotrowska K., Borkowska S.J., Wiszniewska B., Laszczyńska M., Słuczanowska-Głąbowska S., Havens A.M. (2013). The effect of low and high plasma levels of insulin-like growth factor-1 (IGF-1) on the morphology of major organs: studies of Laron dwarf and bovine growth hormone transgenic (bGHTg) mice. Histology & Histopathology.

[10] Hinrichs A., Kessler B., Kurome M., Blutke A., Kemter E., Bernau M. (2018). Growth hormone receptor-deficient pigs resemble the pathophysiology of human Laron syndrome and reveal altered activation of signaling cascades in the liver. Molecular Metabolism.

[11] Wang C., Xu Y. (2019). Mechanisms for sex differences in energy homeostasis. Journal of Molecular Endocrinology.

[12] Robles M.S., Cox J., Mann M. (2014). In-vivo quantitative proteomics reveals a key contribution of post-transcriptional mechanisms to the circadian regulation of liver metabolism. PLoS Genetics.

[13] Mauvoisin D., Wang J., Jouffe C., Martin E., Atger F., Waridel P. (2014). Circadian clock-dependent and -independent rhythmic proteomes implement distinct diurnal functions in mouse liver. Proceedings of the National Academy of Sciences of the U S A.

[14] Wang J., Mauvoisin D., Martin E., Atger F., Galindo A.N., Dayon L. (2017). Nuclear proteomics uncovers diurnal regulatory landscapes in mouse liver. Cell Metabolism.

[15] Albl B., Haesner S., Braun-Reichhart C., Streckel E., Renner S., Seeliger F. (2016). Tissue sampling guides for porcine biomedical models. Toxicologic Pathology.

[16] Blutke A., Renner S., Flenkenthaler F., Backman M., Haesner S., Kemter E. (2017). The Munich MIDY Pig Biobank - a unique resource for studying organ crosstalk in diabetes. Molecular Metabolism.

[17] Rathkolb B., Hans W., Prehn C., Fuchs H., Gailus-Durner V., Aigner B. (2013). Clinical chemistry and other laboratory tests on mouse plasma or serum. Current Protocol Mouse Biology.

[18] Antharavally B.S., Mallia K.A., Rangaraj P., Haney P., Bell P.A. (2009). Quantitation of proteins using a dye-metal-based colorimetric protein assay. Analytical Biochemistry.

[19] Perez-Riverol Y., Csordas A., Bai J., Bernal-Llinares M., Hewapathirana S., Kundu D.J. (2019). The PRIDE database and related tools and resources in 2019: improving support for quantification data. Nucleic Acids Research.

[20] Cox J., Hein M.Y., Luber C.A., Paron I., Nagaraj N., Mann M. (2014). Accurate proteome-wide label-free quantification by delayed normalization and maximal peptide ratio extraction, termed MaxLFQ. Molecular & Cellular Proteomics.

[21] Huang da W., Sherman B.T., Lempicki R.A. (2009). Systematic and integrative analysis of large gene lists using DAVID bioinformatics resources. Nature Protocols.

[22] Subramanian A., Tamayo P., Mootha V.K., Mukherjee S., Ebert B.L., Gillette M.A. (2005). Gene set enrichment analysis: a knowledge-based approach for interpreting genome-wide expression profiles. Proceedings of the National Academy of Sciences of the U S A.

[23] Zukunft S., Sorgenfrei M., Prehn C., Möller G., Adamski J. (2013). Targeted metabolomics of dried blood spot extracts. Chromatographia.

[24] Zukunft S., Prehn C., Rohring C., Moller G., Hrabe de Angelis M., Adamski J. (2018). High-throughput extraction and quantification method for targeted metabolomics in murine tissues. Metabolomics.

[25] Kemter E., Frohlich T., Arnold G.J., Wolf E., Wanke R. (2017). Mitochondrial dysregulation secondary to endoplasmic reticulum stress in autosomal dominant tubulointerstitial kidney disease - UMOD (ADTKD-UMOD). Scientific Reports.

[26] Team R.C. (2018). R: A language and environment for statistical computing.

[27] Wickham H. (2016). ggplot2: elegant graphics for data analysis.

[28] Tyanova S., Temu T., Sinitcyn P., Carlson A., Hein M.Y., Geiger T. (2016). The Perseus computational platform for comprehensive analysis of (prote)omics data. Nature Methods.

[29] Gesing A., Masternak M.M., Wang F., Joseph A.M., Leeuwenburgh C., Westbrook R. (2011). Expression of key regulators of mitochondrial biogenesis in growth hormone receptor knockout (GHRKO) mice is enhanced but is not further improved by other potential life-extending interventions. J Gerontol A Biology Science Medicine Science.

[30] Colnot S., Perret C., Monga S.P.S. (2011). Liver zonation. Molecular pathology of liver diseases.

[31] Karp N.A., Mason J., Beaudet A.L., Benjamini Y., Bower L., Braun R.E. (2017). Prevalence of sexual dimorphism in mammalian phenotypic traits. Nature Communications.

[32] Duran-Ortiz S., Brittain A.L., Kopchick J.J. (2017). The impact of growth hormone on proteomic profiles: a review of mouse and adult human studies. Clinical Proteomics.

[33] Litwack G., Litwack G. (2018). Metabolism of amino acids. Human biochemistry.

[34] Sanderink G.J., Artur Y., Siest G. (1988). Human aminopeptidases: a review of the literature. Journal of Clinical Chemistry & Clinical Biochemistry.

[35] Laron Z. (2004). Laron syndrome (primary growth hormone resistance or insensitivity): the personal experience 1958-2003. Journal of Clinical Endocrinology & Metabolism.

[36] Laron Z., Avitzur Y., Klinger B. (1995). Carbohydrate metabolism in primary growth hormone resistance (Laron syndrome) before and during insulin-like growth factor-I treatment. Metabolism.

[37] Martinez-Reyes I., Chandel N.S. (2017). Waste not, want not: lactate oxidation fuels the TCA cycle. Cell Metabolism.

[38] Baumgart E., Fahimi H.D., Stich A., Volkl A. (1996). L-lactate dehydrogenase A4- and A3B isoforms are bona fide peroxisomal enzymes in rat liver. Evidence for involvement in intraperoxisomal NADH reoxidation. Journal of Biological Chemistry.

[39] Gladden L.B. (2004). Lactate metabolism: a new paradigm for the third millennium. Journal of Physiology.

[40] Newman J.C., Verdin E. (2014). Ketone bodies as signaling metabolites. Trends in Endocrinology and Metabolism.

[41] Rescigno T., Capasso A., Tecce M.F. (2018). Involvement of nutrients and nutritional mediators in mitochondrial 3-hydroxy-3-methylglutaryl-CoA synthase gene expression. Journal of Cellular Physiology.

[42] Grabacka M., Pierzchalska M., Dean M., Reiss K. (2016). Regulation of ketone body metabolism and the role of PPARalpha. International Journal of Molecular Sciences.

[43] Backman M., Flenkenthaler F., Blutke A., Dahlhoff M., Landstrom E., Renner S. (2019). Multi-omics insights into functional alterations of the liver in insulin-deficient diabetes mellitus. Molecular Metabolism.

[44] Sikder K., Shukla S.K., Patel N., Singh H., Rafiq K. (2018). High fat diet upregulates fatty acid oxidation and ketogenesis via intervention of PPAR-gamma. Cellular Physiology and Biochemistry.

[45] Kim J.T., Li C., Weiss H.L., Zhou Y., Liu C., Wang Q. (2019). Regulation of ketogenic enzyme HMGCS2 by wnt/beta-catenin/PPARgamma pathway in intestinal cells. Cells.

[46] Mandard S., Stienstra R., Escher P., Tan N.S., Kim I., Gonzalez F.J. (2007). Glycogen synthase 2 is a novel target gene of peroxisome proliferator-activated receptors. Cellular and Molecular Life Sciences : CMLS.

[47] Longo N., Frigeni M., Pasquali M. (2016). Carnitine transport and fatty acid oxidation. Biochimica et Biophysica Acta.

[48] Flanagan J.L., Simmons P.A., Vehige J., Willcox M.D., Garrett Q. (2010). Role of carnitine in disease. Nutrition and Metabolism (Lond).

[49] Schooneman M.G., Vaz F.M., Houten S.M., Soeters M.R. (2013). Acylcarnitines: reflecting or inflicting insulin resistance?. Diabetes.

[50] Reuter S.E., Evans A.M. (2012). Carnitine and acylcarnitines: pharmacokinetic, pharmacological and clinical aspects. Clinical Pharmacokinetics.

[51] Piccinin E., Cariello M., De Santis S., Ducheix S., Sabba C., Ntambi J.M. (2019). Role of oleic acid in the gut-liver Axis: from diet to the regulation of its synthesis via stearoyl-CoA desaturase 1 (SCD1). Nutrients.

[52] Tateno C., Kataoka M., Utoh R., Tachibana A., Itamoto T., Asahara T. (2011). Growth hormone-dependent pathogenesis of human hepatic steatosis in a novel mouse model bearing a human hepatocyte-repopulated liver. Endocrinology.

[53] Paton C.M., Ntambi J.M. (2009). Biochemical and physiological function of stearoyl-CoA desaturase. American Journal of Physiology. Endocrinology and Metabolism.

[54] List E.O., Berryman D.E., Funk K., Jara A., Kelder B., Wang F. (2014). Liver-specific GH receptor gene-disrupted (LiGHRKO) mice have decreased endocrine IGF-I, increased local IGF-I, and altered body size, body composition, and adipokine profiles. Endocrinology.

[55] Meex R.C., Hoy A.J., Morris A., Brown R.D., Lo J.C., Burke M. (2015). Fetuin B is a secreted hepatocyte factor linking steatosis to impaired glucose metabolism. Cell Metabolism.

[56] Xia M., Liu Y., Guo H., Wang D., Wang Y., Ling W. (2013). Retinol binding protein 4 stimulates hepatic sterol regulatory element-binding protein 1 and increases lipogenesis through the peroxisome proliferator-activated receptor-gamma coactivator 1beta-dependent pathway. Hepatology.

[57] Renner S., Blutke A., Dobenecker B., Dhom G., Müller T.D., Finan B. (2018). Metabolic syndrome and extensive adipose tissue inflammation in morbidly obese Göttingen minipigs. Molecular Metabolism.

[58] Mizuarai S., Miki S., Araki H., Takahashi K., Kotani H. (2005). Identification of dicarboxylate carrier Slc25a10 as malate transporter in de novo fatty acid synthesis. Journal of Biological Chemistry.

[59] Long T., Hassan A., Thompson B.M., McDonald J.G., Wang J., Li X. (2019). Structural basis for human sterol isomerase in cholesterol biosynthesis and multidrug recognition. Nature Communications.

[60] Ji Y., Nordgren K.K., Chai Y., Hebbring S.J., Jenkins G.D., Abo R.P. (2012). Human liver methionine cycle: MAT1A and GNMT gene resequencing, functional genomics, and hepatic genotype-phenotype correlation. Drug Metabolism & Disposition.

[61] Ramani K., Lu S.C. (2017). Methionine adenosyltransferases in liver health and diseases. Liver Research.

[62] Uthus E.O., Brown-Borg H.M. (2003). Altered methionine metabolism in long living Ames dwarf mice. Experimental Gerontology.

[63] Brown-Borg H.M., Rakoczy S.G., Uthus E.O. (2005). Growth hormone alters methionine and glutathione metabolism in Ames dwarf mice. Mechanism of Ageing and Development.

[64] Lu S.C. (2013). Glutathione synthesis. Biochimica et Biophysica Acta.

[65] Mosharov E., Cranford M.R., Banerjee R. (2000). The quantitatively important relationship between homocysteine metabolism and glutathione synthesis by the transsulfuration pathway and its regulation by redox changes. Biochemistry.

[66] Maunoury R., Robine S., Pringault E., Leonard N., Gaillard J.A., Louvard D. (1992). Developmental regulation of villin gene expression in the epithelial cell lineages of mouse digestive and urogenital tracts. Development.

[67] Bacchi C.E., Gown A.M. (1991). Distribution and pattern of expression of villin, a gastrointestinal-associated cytoskeletal protein, in human carcinomas: a study employing paraffin-embedded tissue. Laboratory Investigation.

[68] Rondini E.A., Pant A., Kocarek T.A. (2015). Transcriptional regulation of cytosolic sulfotransferase 1C2 by intermediates of the cholesterol biosynthetic pathway in primary cultured rat hepatocytes. Journal of Pharmacology and Experimental Therapeutics.

[69] Berg J.M., Tymoczko J.L., Stryer L. (2002). Purine bases can Be synthesized de Novo or recycled by salvage pathways. Biochemistry.

[70] Ferris S.P., Kodali V.K., Kaufman R.J. (2014). Glycoprotein folding and quality-control mechanisms in protein-folding diseases. Disease Model Mechcanical.

[71] Jansson J.O., Eden S., Isaksson O. (1985). Sexual dimorphism in the control of growth hormone secretion. Endocrine Reviews.

[72] Wauthier V., Waxman D.J. (2008). Sex-specific early growth hormone response genes in rat liver. Molecular Endocrinology.

[73] Norstedt G., Palmiter R. (1984). Secretory rhythm of growth hormone regulates sexual differentiation of mouse liver. Cell.

[74] Jaffe C.A., Ocampo-Lim B., Guo W., Krueger K., Sugahara I., DeMott-Friberg R. (1998). Regulatory mechanisms of growth hormone secretion are sexually dimorphic. Journal of Clinical Investigation.

[75] Roelfsema F., Veldhuis J.D. (2016). Growth hormone dynamics in healthy adults are related to age and sex and strongly dependent on body mass index. Neuroendocrinology.

