# Supplementary material for: Functional changes of the liver in the absence of growth hormone (GH) action – Proteomic and metabolomic insights from a GH receptor deficient pig model
Source: Mol Metab. 2020 Mar 18;36:100978. doi: 10.1016/j.molmet.2020.100978 (PMC7184181; doi:10.1016/j.molmet.2020.100978)
Supplement: Multimedia component 2 [file mmc2.pdf]

Supplementary Table 2: Addition to Supplementary Figures 1 and 2. List of metabolites measured with the Absolute*IDQ*® p180 Kit GAC.

Acylcarnitines (40)			
C0	Carnitine	C10:1	Decenoylcarnitine
C2	Acetylcarnitine	C10:2	Decadienylcarnitine
C3	Propionylcarnitine	C12	Dodecanoylcarnitine
C3:1	Propenoylcarnitine	C12:1	Dodecenoylcarnitine
C3-OH	Hydroxypropionylcarnitine	C12-DC	Dodecanedioylcarnitine
C4	Butyrylcarnitine	C14	Tetradecanoylcarnitine
C4:1	Butenoylcarnitine	C14:1	Tetradecenoylcarnitine
C4-OH (C3-DC)	Hydroxybutyrylcarnitine	C14:1-OH	Hydroxytetradecenoylcarnitine
C5	Valerylcarnitine	C14:2	Tetradecadienylcarnitine
C5:1	Tiglylcarnitine	C14:2-OH	Hydroxytetradecadienylcarnitine
C5:1-DC	Glutaconylcarnitine	C16	Hexadecanoylcarnitine
C5-DC (C6-OH)	Glutarylcarnitine (Hydroxyhexanoylcarnitine)	C16:1	Hexadecenoylcarnitine
C5-M-DC	Methylglutarylcarnitine	C16:1-OH	Hydroxyhexadecenoylcarnitine
C5-OH (C3-DC-M)	Hydroxyvalerylcarnitine (Methylmalonylcarnitine)	C16:2	Hexadecadienylcarnitine
C6 (C4:1-DC)	Hexanoylcarnitine (Fumarylcarnitine)	C16:2-OH	Hydroxyhexadecadienylcarnitine
C6:1	Hexenoylcarnitine	C16-OH	Hydroxyhexadecanoylcarnitine
C7-DC	Pimelylcarnitine	C18	Octadecanoylcarnitine
C8	Octanoylcarnitine	C18:1	Octadecenoylcarnitine
C9	Nonanoylcarnitine	C18:1-OH	Hydroxyoctadecenoylcarnitine
C10	Decanoylcarnitine	C18:2	Octadecadienylcarnitine
**Amino Acids (21)**			
Ala	Alanine	Lys	Lysine
Arg	Arginine	Met	Methionine
Asn	Asparagine	Orn	Ornithine
Asp	Aspartate	Phe	Phenylalanine
Cit	Citrulline	Pro	Proline
Gln	Glutamine	Ser	Serine
Glu	Glutamate	Thr	Threonine
Gly	Glycine	Trp	Tryptophan
His	Histidine	Tyr	Tyrosine
Ile	Isoleucine	Val	Valine
Leu	Leucine		
**Monosaccharides (1)**			
Sum of Hexoses (including Glucose)			
**Glycerophospholipids (90)**			
lysoPC a C14:0	PC aa C34:1	PC aa C42:0	PC ae C38:2
lysoPC a C16:0	PC aa C34:2	PC aa C42:1	PC ae C38:3

lysoPC a C16:1	PC aa C34:3	PC aa C42:2	PC ae C38:4
lysoPC a C17:0	PC aa C34:4	PC aa C42:4	PC ae C38:5
lysoPC a C18:0	PC aa C36:0	PC aa C42:5	PC ae C38:6
lysoPC a C18:1	PC aa C36:1	PC aa C42:6	PC ae C40:1
lysoPC a C18:2	PC aa C36:2	PC ae C30:0	PC ae C40:2
lysoPC a C20:3	PC aa C36:3	PC ae C30:1	PC ae C40:3
lysoPC a C20:4	PC aa C36:4	PC ae C30:2	PC ae C40:4
lysoPC a C24:0	PC aa C36:5	PC ae C32:1	PC ae C40:5
lysoPC a C26:0	PC aa C36:6	PC ae C32:2	PC ae C40:6
lysoPC a C26:1	PC aa C38:0	PC ae C34:0	PC ae C42:0
lysoPC a C28:0	PC aa C38:1	PC ae C34:1	PC ae C42:1
lysoPC a C28:1	PC aa C38:3	PC ae C34:2	PC ae C42:2
PC aa C24:0	PC aa C38:4	PC ae C34:3	PC ae C42:3
PC aa C26:0	PC aa C38:5	PC ae C36:0	PC ae C42:4
PC aa C28:1	PC aa C38:6	PC ae C36:1	PC ae C42:5
PC aa C30:0	PC aa C40:1	PC ae C36:2	PC ae C44:3
PC aa C30:2	PC aa C40:2	PC ae C36:3	PC ae C44:4
PC aa C32:0	PC aa C40:3	PC ae C36:4	PC ae C44:5
PC aa C32:1	PC aa C40:4	PC ae C36:5	PC ae C44:6
PC aa C32:2	PC aa C40:5	PC ae C38:0	
PC aa C32:3	PC aa C40:6	PC ae C38:1	
**Sphingolipids (15)**			
SM (OH) C14:1	SM C18:0	SM (OH) C22:1	SM (OH) C24:1
SM C16:0	SM C18:1	SM (OH) C22:2	SM C26:0
SM C16:1	SM C20:2	SM C24:0	SM C26:1
SM (OH) C16:1	SM C22:3	SM C24:1	
**Biogenic Amines (21)**			
Ac-Orn	Acetylornithine	PEA	Phenylethylamine
ADMA	Asymmetric dimethylarginine	*cis*-OH-Pro	*cis*-4-Hydroxyproline
alpha-AAA	alpha-Aminoadipic acid	*trans*-OH-Pro	*trans*-4-Hydroxyproline
Carnosine	Carnosine	Putrescine	Putrescine
Creatinine	Creatinine	SDMA	Symmetric dimethylarginine
DOPA	DOPA	Serotonin	Serotonin
Dopamine	Dopamine	Spermidine	Spermidine
Histamine	Histamine	Spermine	Spermine
Kynurenine	Kynurenine	Taurine	Taurine
Met-SO	Methionine sulfoxide	total DMA	Total dimethylarginine
Nitro-Tyr	Nitrotyrosine		
