# Supplementary material for: Functional changes of the liver in the absence of growth hormone (GH) action – Proteomic and metabolomic insights from a GH receptor deficient pig model
Source: Mol Metab. 2020 Mar 18;36:100978. doi: 10.1016/j.molmet.2020.100978 (PMC7184181; doi:10.1016/j.molmet.2020.100978)
Supplement: Multimedia component 7 [file mmc7.pdf]

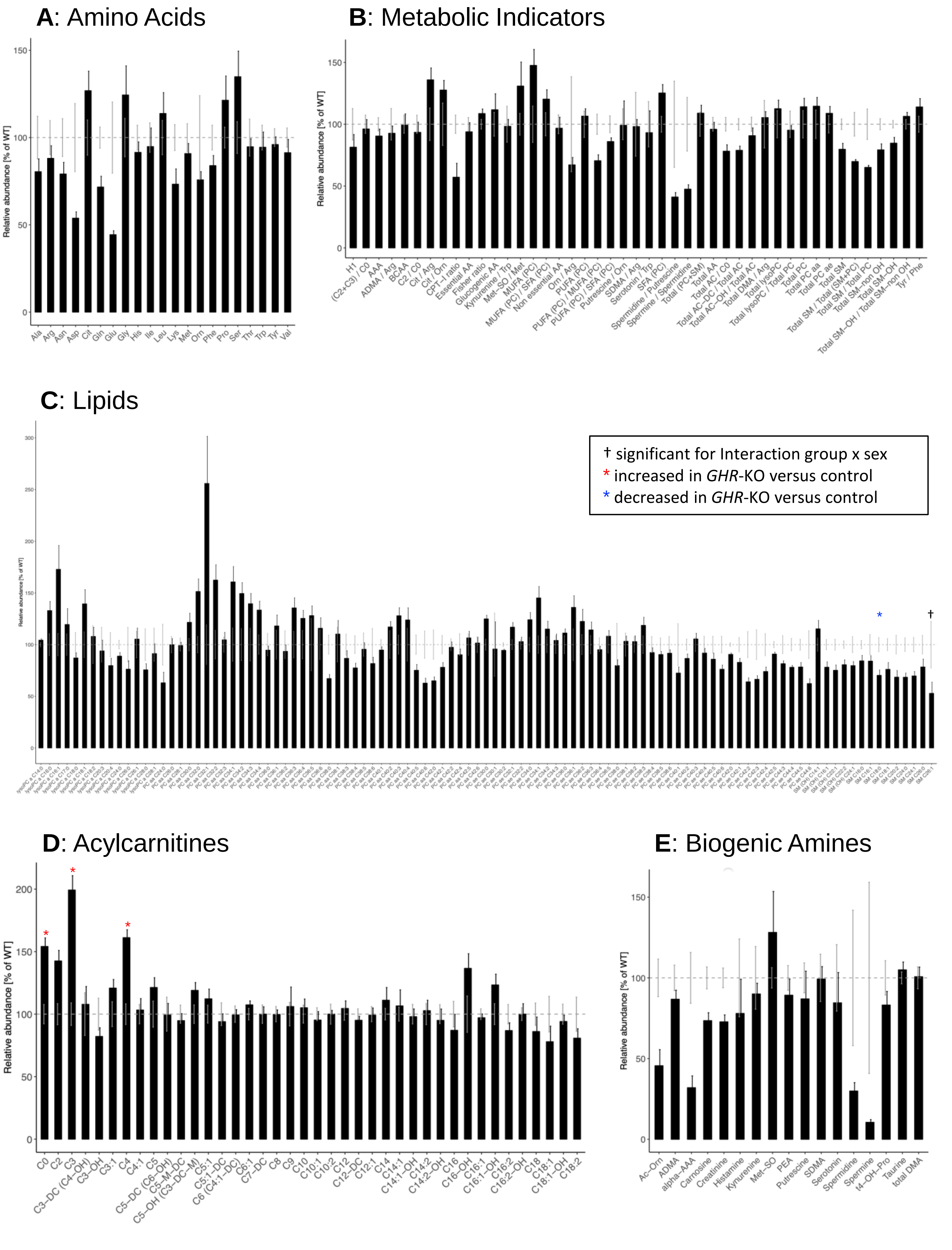

Supplementary Figure 1: Relative abundance changes of metabolites in *GHR*-KO and control serum samples. Graphs show the relative abundances of amino acids (A), metabolic indicators (B), lipids (C), acylcarnitines (D), and biogenic amines (E) determined by targeted metabolomics. The dashed line indicates the mean abundance of the control samples, bars show the mean relative abundance in *GHR*-KO samples. Error bars represent the standard error of the mean (SEM).

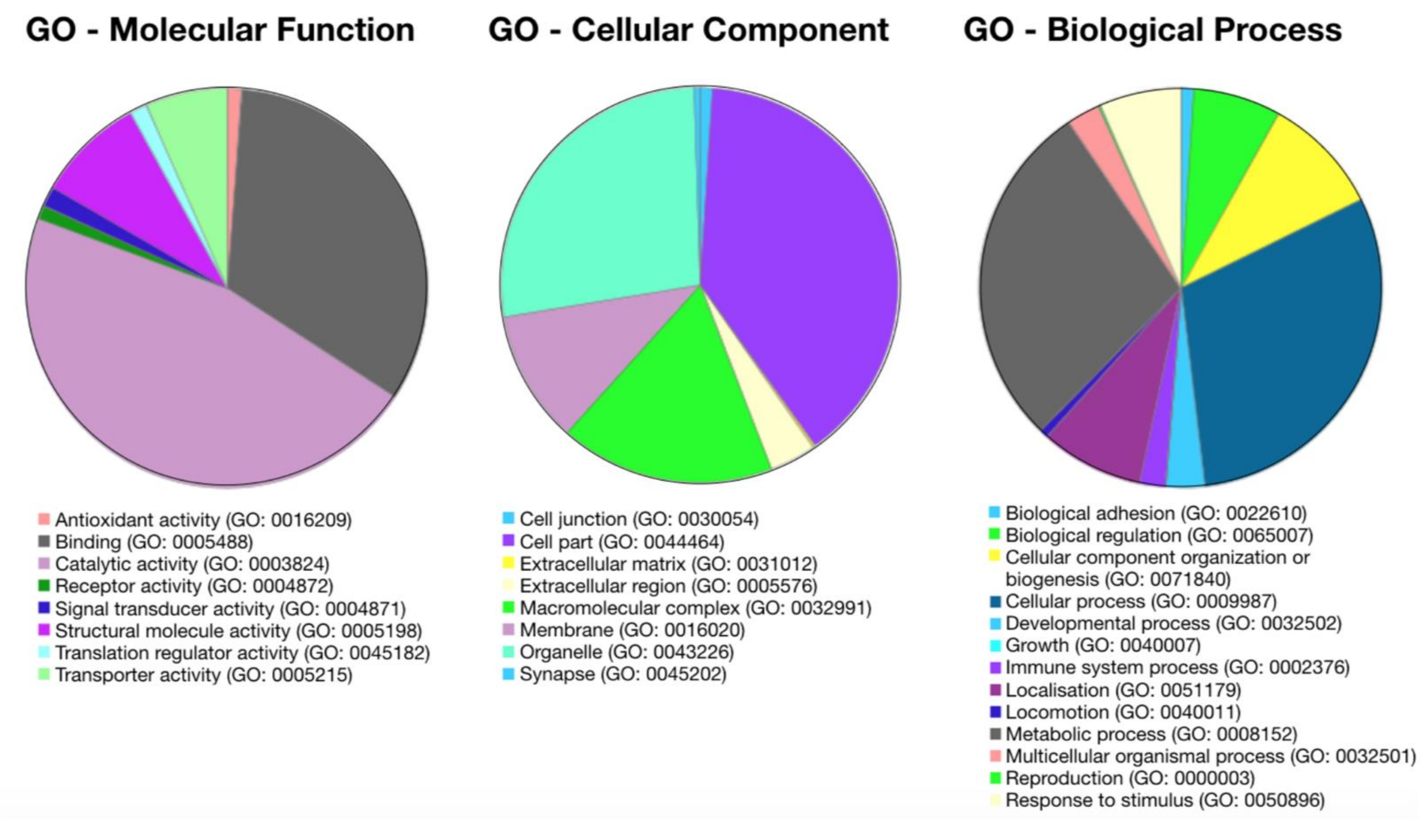

Supplementary Figure 2: GO-Analysis of the 3232 detected protein families (see also Supplementary Table 1A) measured with QExactive (Thermo Scientific) using PANTHER (pantherdb.org).

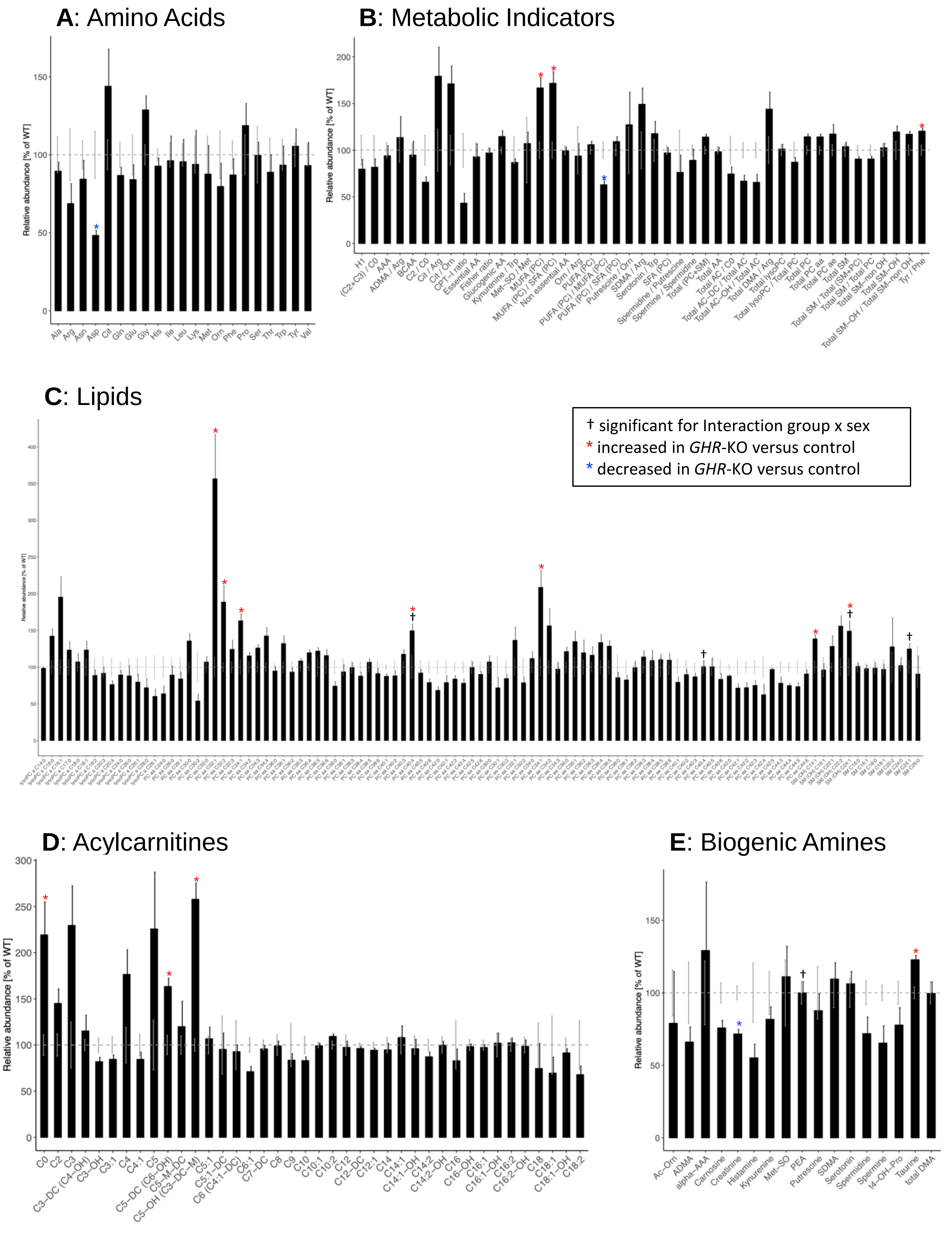

Supplementary Figure 3: Relative abundance changes of metabolites in *GHR*-KO vs. control liver tissue. Graphs show the relative abundances of amino acids (A), metabolic indicators (B), lipids (C), acylcarnitines (D), and biogenic amines (E) determined by targeted metabolomics. The dashed line indicates the mean abundance of the control samples, bars show the mean relative abundance in *GHR*-KO samples. Error bars represent the standard error of the mean (SEM).
